# Reduced urine volume and changed renal sphingolipid metabolism in P2ry14-deficient mice

**DOI:** 10.3389/fcell.2023.1128456

**Published:** 2023-05-11

**Authors:** Fabian Baalmann, Jana Brendler, Anne Butthof, Yulia Popkova, Kathrin M. Engel, Jürgen Schiller, Karsten Winter, Vera Lede, Albert Ricken, Torsten Schöneberg, Angela Schulz

**Affiliations:** ^1^ Rudolf Schönheimer Institute of Biochemistry, Faculty of Medicine, Leipzig University, Leipzig, Germany; ^2^ Institute of Anatomy, Faculty of Medicine, Leipzig University, Leipzig, Germany; ^3^ Institute of Medical Physics and Biophysics, Faculty of Medicine, Leipzig University, Leipzig, Germany

**Keywords:** P2RY14, kidney, papilla, intercalated cells, aquaporin-2, sphingolipids

## Abstract

The UDP-glucose receptor P2RY14, a rhodopsin-like G protein-coupled receptor (GPCR), was previously described as receptor expressed in A-intercalated cells of the mouse kidney. Additionally, we found P2RY14 is abundantly expressed in mouse renal collecting duct principal cells of the papilla and epithelial cells lining the renal papilla. To better understand its physiological function in kidney, we took advantage of a P2ry14 reporter and gene-deficient (KO) mouse strain. Morphometric studies showed that the receptor function contributes to kidney morphology. KO mice had a broader cortex relative to the total kidney area than wild-type (WT) mice. In contrast, the area of the outer stripe of the outer medulla was larger in WT compared to KO mice. Transcriptome comparison of the papilla region of WT and KO mice revealed differences in the gene expression of extracellular matrix proteins (e.g., decorin, fibulin-1, fibulin-7) and proteins involved in sphingolipid metabolism (e.g., small subunit b of the serine palmitoyltransferase) and other related GPCRs (e.g., GPR171). Using mass spectrometry, changes in the sphingolipid composition (e.g., chain length) were detected in the renal papilla of KO mice. At the functional level, we found that KO mice had a reduced urine volume but an unchanged glomerular filtration rate under normal chow and salt diets. Our study revealed P2ry14 as a functionally important GPCR in collecting duct principal cells and cells lining the renal papilla and the possible involvement of P2ry14 in nephroprotection by regulation of decorin.

## Introduction

P2RY14 is one of the metabotropic P2 nucleotide receptors belonging to the class of rhodopsin-like G protein-coupled receptors (GPCRs). More than 20 years ago, P2RY14 (former GPR105, KIAA0001) was deorphanized and described as a receptor for UDP-glucose and other sugar derivatives (Chambers et al., 2000). As other related members of the P2RY12-like group, P2RY14 transduces extracellular signals through the G_i_ subclass of heterotrimeric G proteins (Chambers et al., 2000; Schöneberg et al., 2007; Carter et al., 2009). In addition to the G_i_-mediated inhibition of adenylyl cyclase activity, receptor-mediated ERK1/2 phosphorylation was observed (Carter et al., 2009).

Previous research on P2RY14 showed widespread expression and implication in many physiological processes and diseases. P2RY14 is expressed in many organs and tissues including placenta, spleen, thymus, lung, heart, brain, kidney, gastrointestinal tract, pancreas, and adipose tissue (Chambers et al., 2000; Freeman et al., 2001; Meister et al., 2014). In those tissues, P2RY14 expression is found in different immune and non-immune cells (for review see Lazarowski und Harden, 2015). Its physiological functions may include modulations of immune responses (Skelton et al., 2003; Scrivens und Dickenson, 2005; Arase et al., 2009; Barrett et al., 2013; Gendaszewska-Darmach et al., 2016; Karcz et al., 2021), hematopoietic stem cell localization and senescence (Lee et al., 2003; Cho et al., 2014), modulation of smooth muscle tone (Bassil et al., 2009; Haanes et al., 2014; Meister et al., 2014), glucose homeostasis (Xu et al., 2012; Meister et al., 2014), and bone metabolism (Lee et al., 2013; Mikolajewicz und Komarova, 2020). In addition, P2RY14 expression was also found in different cancers suggesting P2RY14 as potential biomarker for specific neoplasms (Shah et al., 2018; Li et al., 2021; Patritti-Cram et al., 2021).

Previously, P2ry14 expression was shown in kidney, specifically in intercalated cells type A, being involved in proinflammatory responses (Azroyan et al., 2015; Chen et al., 2017; Battistone et al., 2020). Thus, receptor inactivation with a specific antagonist or gene knockout was found to be protective against acute kidney injury in a mouse model of ischemia reperfusion injury (Battistone et al., 2020). To further understand its physiological renal function, we took advantage of a P2ry14 reporter and gene-deficient mouse strain. Here, we report that P2ry14 is highly expressed in the renal papilla. Transcriptome, biochemical and functional studies with P2ry14-deficient mice revealed its function in renal morphology, renal sphingolipid metabolism and urine volume determination.

## Material and methods

### Animals

P2ry14-deficient mice were generated at TAKEDA Cambridge Ltd. as described previously (Meister et al., 2014). Briefly, the coding region of the receptor was replaced by a lacZ expression cassette allowing galactosidase staining of cells endogenously expressing P2ry14 (Meister et al., 2014). Backcrossing for more than 20 generations into the C57BL/6J background generated the mouse line used in this study. The mice were kept under specific-pathogen-free conditions on a 12-h light/12-h dark cycle with *ad libitum* access to food and water. Experiments were performed with littermates from heterozygous parents and according to the accepted standards of animal care. Approval was given by the respective regional government agency of the State of Saxony, Germany (T46/12, T07/13, T21/18, TVV20/20). Mice were fed a regular chow or a salt (NaCl)-enriched diet (4% NaCl) (from Sniff Spezialitäten GmbH, Soest, Germany) (for composition see Supplementary Table S1). Most experiments were performed with mice from both sexes. If no significant sex-specific differences were found in initial experiments, final data sets were generated from only one sex, which is given in the respective method section.

### X-Gal and SPiDER-Gal staining of kidney cryosections

Kidneys from wildtype (WT) and knockout (KO) mice removed at postnatal day (pd) 1, pd 4, pd 14, pd 21, and from adult mice (8–12 weeks) were embedded in TissueTek^®^ (Sakura, Torrance, CA, United States) and shock-frozen with liquid nitrogen. The tissue was cryosectioned. Ten µm thick sections were fixed in ice cold methanol/acetone (1:1, v/v) for 1 min and air-dried for 15 min. For X-Gal staining the sections were incubated in an HEPES-buffered X-Gal staining solution (pH 7.5) at 37°C overnight to detect β-galactosidase activity (Merkwitz et al., 2016). For confocal microscopy β-galactosidase activity was visualized using SPiDER-Gal (tebu-bio GmbH, Offenbach a. M., Germany) according to the manufacturer’s instructions. Briefly, SPiDER-Gal stock solution was prepared according to the manufacturer’s protocol. Sections were incubated with a 1:1000 dilution of the stock solution in HBSS buffer for 15 min at 37°C. Aquaporin-2 (Aqp2) staining of collecting duct cells was performed with a 1:100 dilution of anti-Aqp2-antibody (ab199975; Abcam, Cambridge, UK) and alexa-fluor 568 antibody (a-11036, 1:500, Thermo Fisher, Schwerte, Germany) as secondary antibody. For nucleus staining a 300 nM 4′,6-diamidin-2-phenylindol (DAPI) solution in PBS was used.

### Fluorescence *in situ* hybridization

The RNAscope Multiplex Fluorescent Reagent Kit v2 (Advanced Cell Diagnostics [ACD], Berlin, Germany) was used to colocalize multiple RNAs on the same section. Isolated kidney halves with papillae were fixed with 4% formaldehyde for 24 h and embedded in paraffin wax. Ten µm thick sections were cut from the paraffin blocks and mounted on Superfrost^®^ slides. For triple labeling, mixtures were prepared from 1:50 diluted commercially purchased probes from ACD for P2ry14 (REF: 450251), aquaporin-2 (REF: 452411-C3) and either Gpr116 (REF: 318021-C2) or pendrin (452491-C2). The freshly prepared mixtures were incubated on the sections for 2 h at 40°C in a HybEZ™ II manual assay hybridization system (ACD). Three consecutive amplification steps and labeling steps followed using single Opal fluorophores at dilutions of 1:750 (Akoya Biosciences, Marlborough, MA) according to manufacturer’s instructions. P2ry14 C1-RNA was labeled with Opal 520 (OP-001001), aquaporin-2 C3-RNA was labeled with Opal 570 (OP-001003) and either Gpr116 or pendrin C2-RNA was labeled with Opal 690 (OP-001006). Between each RNA labeling step, sections were thoroughly washed with the wash buffer included in the kit. After labeling, DAPI nuclear counterstaining (Serva, Heidelberg, Germany) was performed and sections were embedded in Dako fluorescent embedding medium (Aligent, Frankfurt, Germany). The LSM 700 confocal laser-scanning microscope from Zeiss (Jena, Germany) was used to evaluate the obtained fluorescence signal*.* To assess the background or non-specific staining in conjunction with the RNAscope target probes used in this study, positive and negative probes from the manufacturer were used for kidney sections as technical controls. The negative probe was specific to bacterial D-box binding PAR bZIP transcription factor (dabP) mRNA and the positive control probes were specific to mouse RNA polymerase II subunit A mRNA (Polr2a (Opal 520; green), peptidylprolyl isomerase B mRNA (Ppib (Opal 570; red) and ubiquitin C mRNA (Ubc (Opal 690; white). Results are shown in Supplementary Figure S1C.

### Morphometric analysis

Fractionated series of ten µm thick, formalin-fixed embedded WT and KO tissue sections were used. Mid-organ slices were stained with Alcian blue and Periodic-acid Schiff reagents. After manual determination of the different kidney regions, the sections were fully digitized at 20× magnification using a digital slide scanner (Pannoramic Scan II, 3D HISTECH Ltd., Budapest, Hungary) and exported (CaseViewer, Version 2.3, 3D HISTECH Ldt., Budapest, Hungary) as images with a pixel size of 0.24 µm. The different areas and stripes of the kidney [cortex, outer stripe of the outer medulla (OSOM), inner stripe of the outer medulla (ISOM) and inner medulla/papilla (IM/P)] as well as all glomeruli were highlighted manually using GIMP (Version 2.10.2, The GIMP team, http://www.gimp.org) (Supplementary Figure S2). The area of these compartments was measured and the number of glomeruli was automatically counted using Mathematica (Version 11.3, Wolfram Research Inc., Champaign, IL, United States). Ratios of specific layers and total kidney area and glomeruli per area of cortex and total kidney were calculated and compared between WT and KO (n = 18 sections from 11 mice per group, left and right kidney of mice). Sections with poor quality were excluded from further analysis (4 sections per group).

### Quantitative expression analysis (RT-qPCR)

Kidneys were removed from WT and KO mice. For expression analysis, samples of cortex, medulla and papilla were prepared under a stereomicroscope. Tissue samples were immersed with TRIzol (Sigma-Aldrich, Merck, Darmstadt, Germany), homogenized with a Precellys^®^ 24 homogenizer (Bertin Technologies SAS, Montigny-le-Bretonneux, France) and stored at −80°C until RNA preparation. Thawed homogenate was mixed with 300 µl chloroform and centrifuged for 5 minutes at 13,000 rpm. The aqueous phase was mixed with one volume of 70% ethanol and transferred to an RNeasy spin column (Qiagen, Hilden, Germany). After centrifugation and washing with buffers according to the manufacturer’s instructions, RNA was eluted with RNase-free water. The amount of RNA was measured using a spectrophotometer (Nanodrop ND 1000; PEQLAB Biotechnologie GmbH, Erlangen, Germany). One microgram RNA was reverse transcribed with the Omniscript RT Kit (Qiagen) using mixed random hexamers and oligo (dT) primers. Quantitative PCR (qPCR) was performed with GoTaq^®^ qPCR Master Mix (Promega, Walldorf, Germany) on a BioRad CFX Connect Real-Time-System (Bio-Rad Laboratories GmbH, Feldkirchen, Germany). The following protocol was used: 95°C for 2 min, 40 cycles of 95°C for 15 s, 63°C for 30 s and 95°C for 10 s. Prior to qPCR, primers were tested in a gradient PCR to determine the optimal annealing temperature. A melt curve was recorded to check for the presence of a single product. ΔCq values were calculated using mouse β2-microglobulin as the reference gene. Primers used for amplification were designed spanning introns to exclude false Cq values due to contamination with genomic DNA (primer sequences are given in Supplementary Table S2).

### Metabolic cages

To quantify food and water intake and excretion, we used a metabolic cage system (TSE Systems, Bad Homburg, Germany). Eight mice were monitored in parallel in cages with *ad libitum* access to food and water for 72 h. Urine and feces were collected after 24, 48, and 72 h and analyzed afterwards. Food that accidentally fell into the collecting container was weighed and considered in the determination of the food intake. To detect individual changes in kidney function, mice were first monitored under chow diet. Subsequently, the diet was changed to a salt-enriched diet for 5 days before mice were again monitored. Five independent experiments were conducted with three to five mice per genotype. Osmolality of the collected urine samples from chow diet-fed mice was determined with a vapor pressure osmometer 5600 (Kreienbaum Neoscience GmbH, Langenfeld, Germany). To determine the osmolality of mouse urine samples within the linear range of the instrument, the samples were diluted with distilled water.

### Relative Inulin-FITC clearance

For comparison of the kidney’s filtrating function between of WT and KO mice, the free filterable molecule inulin was used. The clearance of fluorescent-labeled inulin (inulin-FITC) was determined using a protocol described previously (Qi et al., 2004; Qi und Breyer, 2009). After anesthetizing mice with 100 mg ketamine/10 mg xylazine per kg body weight administered intraperitoneally and obtaining a baseline blood sample at the tail, mice were injected retroorbitally with dialyzed and diluted inulin-FITC (TdB Labs, Uppsala, Sweden). Blood samples were collected in hematocrit capillaries at 2, 4, 6, 8, 10, 15, and 30 min after injection of inulin-FITC. Mice were kept under anesthesia during the procedure and finally sacrificed by cervical dislocation. Samples were centrifuged in a hematocrit centrifuge (Hettich Zentrifugen, Tuttlingen, Germany) for 2 min to obtain plasma. Fluorescence measurement of plasma samples was performed with an Envision Multiplate Reader (Perkin Elmer, Rodgau, Germany) at 525 nm. We used the decline of fluorescence as a surrogate for inulin-FITC clearance from blood. Fluorescence at 2 min after injection was set as maximal fluorescence = 100%. Fluorescence at the following time points was calculated as the percentage of maximal fluorescence. Samples with inadequately decreasing fluorescence (relative fluorescence at 30 min not below 67% of maximal fluorescence) were excluded.

### Blood pH and papillary cAMP measurement

Blood pH was determined with an ABL90 Flex instrument (Radiometer GmbH, Krefeld, Germany). Mice were anesthetized with ketamine/xylazine (see above), blood was taken retroorbitally with a 65-µl capillary and blood parameters were measured according to manufacturer’s instructions. Then, mice were sacrificed by cervical dislocation and kidneys were removed. The papilla was prepared and collected in cAMP lysis buffer. After homogenization of the tissue with a Precellys^®^ 24 homogenizer, the protein content of the papilla samples was determined with the Pierce BCA Protein Assay Kit (Thermo Scientific). For cAMP concentration measurements, the AlphaScreen cAMP detection kit (Perkin Elmer) was used according to the manufacturer’s instructions. The relation between protein content and cAMP concentration of the samples was calculated to compare the samples of WT and KO papillae.

### RNA sequencing of kidney papillae

Female WT and KO mice were fed either chow or salt-enriched diet. Using the protocol described above for quantitative expression analysis total RNA was extracted from papillae of n = 10 mice per group (WT/chow; KO/chow; WT/salt; KO/salt). RNA quantity was measured using a spectrophotometer (Nanodrop ND 1000). The quality of all samples was examined on the Agilent 2100 bioanalyzer (Agilent Technologies, Santa Clara, CA, United States) using the RNA 6000 Nano Chip. Only RNA samples with a RNA integrity number (RIN) above 8 were included.

Indexed cDNA libraries were generated using a TruSeq Stranded mRNA Library Prep Kit (Illumina, Eindhoven, Netherlands) according to the manufacturer’s protocol. Constructed libraries had an average size of 250–300 bp as evaluated on the Agilent 2100 bioanalyzer with a DNA 1000 Chip. Libraries were sequenced on an Illumina HiSeq 4000 with ten biological replicates per genotype. Raw paired-end reads with 101 bp were generated with 10 samples per single flow cell lane (Macrogen, Seoul, South Korea).

### Data analysis

The raw data underwent a quality check using FastQC to verify base calling correctness. Due to poor quality, the first three bases of forward-oriented reads had to be trimmed using Seqtk software (https://github.com/lh3/seqtk). The paired-end reads were mapped to a mouse reference genome (GRCm38/mm10) with TopHat (version 2.0.14) which uses BoWTie (version 1.1.2) for read alignment. Cufflinks (version 2.2.1) was used to calculate values of fragments per kilobase of transcript per million mapped reads (FPKM). Differential expression analysis was performed in R version 3.4.1 (2017-06-30, Core Team (2017). R: A language and environment for statistical computing. R Foundation for Statistical Computing, Vienna, Austria. URL https://www.R-project.org/) using the DESeq2 software package (Love et al., 2014). It was conducted including all genes and after excluding genes with FPKM cut off values < 1 in at least half of the samples. Differentially expressed genes with a *p*-value < 0.05 were considered statistically significant. The sequences for this project were loaded into BioProject (ID: PRJNA923283).

## Lipid measurements in papilla samples

### Lipid extraction

Tissue samples were extracted using MMC (MeOH/MTBE/CHCl_3_) method according to (Pellegrino et al., 2014). Briefly, tissue samples were transferred into ball mill tubes (Precellys^®^ ceramic-kit, CK14, Bertin GmbH, Frankfurt am Main, Germany) containing 665 µl methanol containing 1 mM *t*-butylhydroxytoluene (BHT) preventing oxidation and homogenized using the tissue homogenizer Precellys^®^ 24 (PEQLAB) for 2 × 15 s at 5,000 rpm and put on ice immediately. After transfer of the homogenized samples into glass vials, ball mill tubes were washed with 665 µl methanol (containing 1 mM BHT) to reach a total volume of 1.33 ml; afterwards, 2 ml of MTBE/chloroform (1:1, v/v) were added to the samples. After incubation for 1 h and shaking at 950 rpm at room temperature using a thermoblock, samples were centrifuged for 10 min at 2,000 rpm. The upper phase was transferred to a glass vial. To maximize the extraction yields, the pellet was subjected to a second extraction step with MMC mixture (MeOH/MTBE/CHCl_3_, 1.33:1:1, v/v/v). The obtained organic phases were combined and supernatant was removed by evaporation under a gentle nitrogen steam. The samples were stored at −20°C until further experiments.

### High-performance thin-layer chromatography electrospray ionization ion trap mass spectrometry (HPTLC ESI-IT MS)

HPTLC and ESI-IT MS measurements were performed according to Engel et al. (2017) with slight modifications. Lipid extracts were dissolved to give 10 μg/μl solutions in chloroform and 10 μl of each sample were sprayed onto an HPTLC silica gel 60 plate (Merck KGaA, Darmstadt, Germany) using a CAMAG^®^ Linomat 5 semi-automatic sample application system (CAMAG, Berlin, Germany). Plates were developed in vertical TLC chambers with chloroform/ethanol/water/triethylamine (30:35:7:35, v/v/v/v) as the mobile phase. Lipids were visualized by dipping the entire plate in primuline (Direct Yellow 59, Sigma-Aldrich, Taufkirchen, Germany) dissolved in acetone/water (80:20, v/v, 50 mg/l). Upon illumination with UV light (366 nm), lipids were detected as colored spots. Sphingomyelin (SM) fractions were pencil-marked and automatically eluted by a Plate Express™ TLC plate reader (Advion, Ithaca, NY, United States) with methanol as the solvent and subsequently directly infused into the ESI mass spectrometer.

ESI-IT MS was performed on an Amazon SL mass spectrometer (Bruker Daltonics GmbH, Bremen, Germany). The following conditions were used: spray voltage 4.5 kV, end plate offset 500 V, nebulizer gas 7.3 psi, drying gas (N_2_) 3 l/min, capillary temperature 180°C, flow rate 3 μl/min, sheath gas (He) flow rate 25 a.u. The spectra were recorded in the positive ion mode with enhanced resolution. For data acquisition and subsequent analysis, the Bruker Trap Control and Data Analysis version 4.1 software (Bruker Daltonics GmbH) were used, respectively.

### Statistics

Statistical analyses were performed in GraphPad Prism (Version 6.05 for Windows, GraphPad Software, La Jolla, CA, United States). After testing for normality using the Shapiro-Wilk test samples were compared using t-test. For non-parametric statistical analyses the Mann-Whitney-U-test was used instead.

## Results

### P2ry14 is mainly expressed in principal cells of the mouse renal papilla

**FIGURE 1:**
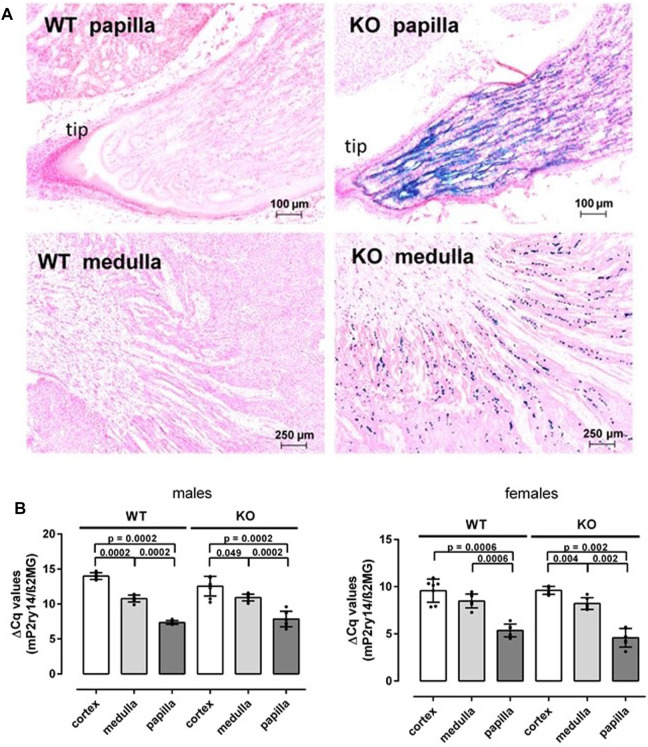
Renal P2ry14 expression is found in medulla and in the papilla. **(A)** Frozen sections of adult WT and KO kidneys were stained with X-Gal and counterstained with nuclear-fast red-aluminum salt. Representative regional sections of papilla and medulla are shown. ß-galactosidase-positive cells were found at the tip of the KO renal papilla and the remaining medulla. No X-Gal staining was observed in WT controls. **(B)** Quantitative PCR reactions were performed with cDNA generated from RNA of cortex, medulla and papilla tissue, collected from males and females of WT and P2ry14-deficient mice. ß2-microglobulin was used as housekeeping gene and gene expression is shown as ΔCq values. Expression of P2ry14 is markedly higher (lower ΔCq values) in the renal papilla compared to medulla and cortex. No significant difference in expression is found between WT and KO animals but a higher P2ry14 expression could be detected in females compared to males. Note, we used primers annealing to 5′-UTR which is not altered by the knockout/knockin procedure (see *Materials and Methods*). WT males: n = 8; KO males: n = 8; WT females n = 7, KO females: n = 7. Data are given as mean ± SD and *p* values were calculated with Mann-Whitney-U-test.

To study the cellular expression of P2ry14 in the mouse kidney, we took advantage of the lacZ expression cassette replacing the P2ry14 coding exon and generating a knockout allele. As previously investigated, bacterial ß-galactosidase expression and, hence, intensity of X-Gal staining is regulated by the P2ry14 promoter and directly reflects the endogenous expression of P2ry14 in WT mouse kidneys (Meister et al., 2014). Most abundant X-Gal staining was observed in the kidney papilla (Figure 1A). The staining intensity increased towards the tip of the papilla. Additional staining was detected in the outer medulla. However, only single cells dispersed in this layer showed ß-galactosidase activity (Figure 1A). This suggested that the papilla also expresses P2ry14 within the mouse kidney in significant amounts. To validate the results of local X-Gal staining, we quantified expression of P2ry14 and the KO construct using RT-qPCR. The gene expression was analyzed in cortex, medulla and papilla from the same kidney samples of both female and male mice. To rule out expression changes due to the insertion of the lacZ reporter cassette in knockout animals, we used primers annealing to 5′-UTR exons, which are not altered by the knockout/knockin procedure. This allowed comparing transcript levels irrespective of the genotype. The papilla showed significantly higher expression of P2ry14 compared to cortex and medulla in both sexes (Figure 1B). Importantly, the gene expression levels in renal medulla and papilla were not different between KO and WT mice. In general, we detected higher expression levels of P2ry14 in females tissues (Figure 1B).

**FIGURE 2:**
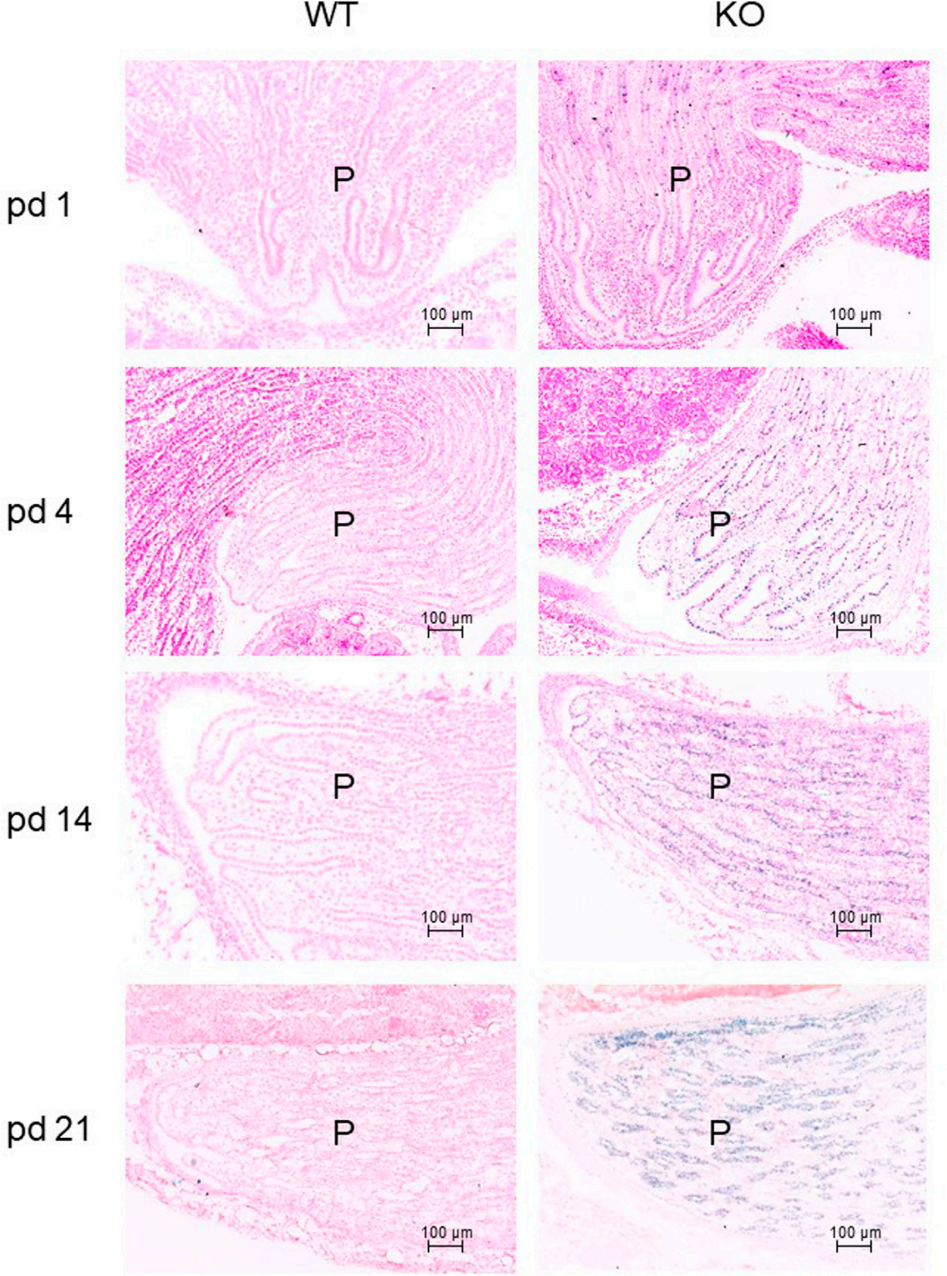
Expression of P2ry14 during postnatal kidney development. Frozen sections of postnatal renal papillae were stained with X-Gal to illustrate ß-galactosidase expression during kidney development between pd 1 and pd 21. Only a few cells express the reporter gene in pd 1 KO animals. During kidney maturation, the ß-galactosidase expression is significantly increased in the sections of KO animals. WT sections are shown for comparison (WT pups n = 2–3; KO pups n = 3–5; at particular times). P: papilla.

The development of the mouse kidney is not completed at birth (McMahon, 2016). Therefore, we examined P2ry14 expression during postnatal development using X-Gal staining of kidney sections. Thus, sections of WT (control) and KO mice at pd 1, 4, 14, and 21 were stained (Figure 2). Only a few cells were X-Gal-positive in pd 1 sections of KO animals. However, the number of X-Gal-positive cells increased in the renal papilla within the following days and weeks after birth.

**FIGURE 3:**
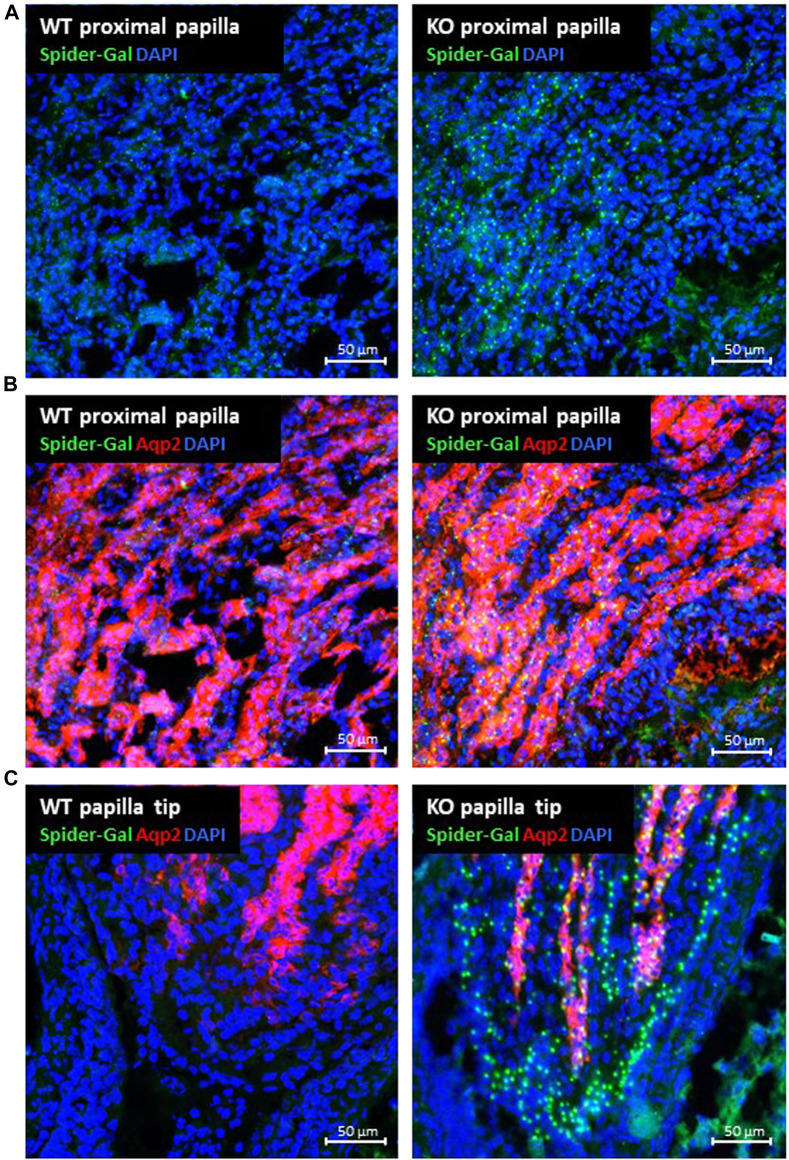
Expression of P2ry14 and colocalization with aquaporin 2 in renal papillae. Frozen sections with renal papillae of WT (control) and KO animals were incubated with the fluorescent ß-galactosidase substrate SPiDER-Gal (tebu-bio GmbH) (green fluorescence) **(A–C)**. Renal collecting duct principal cells were identified using an anti-aquaporin-2 (Aqp2) antibody (ab 199975; abcam) (red) **(B,C)**. As found with X-Gal, collecting duct cells in the papillar region expressed P2ry14. At the tip of the renal papilla **(C)** a substantial number of Aqp2-negative cells in the terminal parts of the collecting ducts and in the epithelium lining the tip of the renal papilla expressed P2ry14 in addition.

Next, we determined the P2ry14-expressing cell type within the papilla. Thus, we used the fluorescent dye SPiDER-Gal, a ß-galactosidase substrate being trapped within the cell after enzymatic hydrolysis. As expected, SPiDER-Gal staining showed β-galactosidase expression similar to X-Gal staining in the papilla region (Figure 3A). Counterstaining with an anti-Aqp2 antibody assigned the SPiDER-Gal signal to collecting duct principal cells (Figure 3B). As shown in Figure 3C, in the papilla, in addition to SPiDER-Gal/Aqp2-positive collecting duct principal cells, a fraction of SPiDER-Gal-positive cells corresponded to Aqp2-negative epithelial cells in the terminal parts of the collecting ducts and in the epithelial lining of the renal papilla.

**FIGURE 4:**
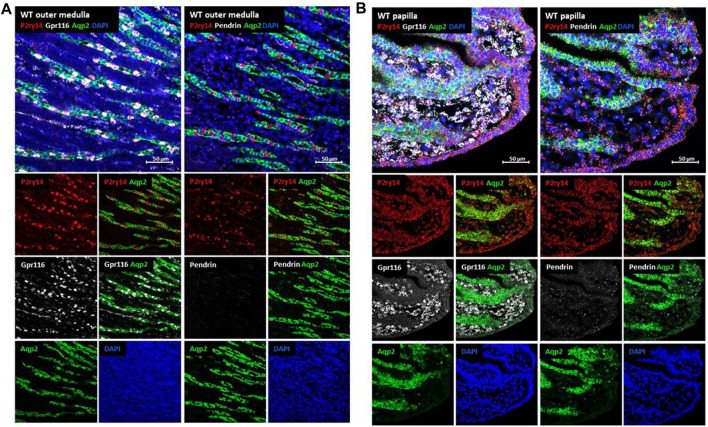
Analysis of P2ry14 expressing cells in the kidney by *in-situ* hybridization. For verification of P2ry14 expression in type A-ICs in the outer medulla and in principal cells of the renal papilla of mice *in-situ* hybridization with WT tissue was performed. To differentiate type A- and type B-ICs from collecting duct principal cells, tissues were co-stained with probes for Gpr116 (A-ICs), pendrin (B-ICs) and Aqp2 (collecting duct principal cells). In the renal outer medulla **(A)**, P2ry14-positive signals (red) were found exclusively in Gpr116-positive cells (white, left side) indicating expression in type A-ICs. No pendrin-positive cells were noted (missing white signal, right side) supporting the finding of type B-ICs only in cortex of mouse kidneys. In WT renal papillae **(B)** a strong P2ry14-positive signal was detected. Here, P2ry14 colocalizes with Aqp2-positive collecting duct principal cells and epithelial cells lining the tip of the renal papilla.

Because commercially available antibodies against P2ry14 failed to convincingly identify P2ry14 positive cells in WT compared to KO mice, we focused on *in-situ* hybridization to further characterize the cell populations expressing P2ry14. Azroyan et al. showed P2ry14 expression only in type A intercalated cells (A-ICs) of the medulla using a mouse model expressing EGFP under control of the V-ATPase promoter (Azroyan et al., 2015). Our data from X-Gal and SPiDER-Gal experiments (see above) in KO animals indicate that P2ry14 expression is not limited to intercalated cells. To rule out the possibility that the co-expression of P2ry14 with Aqp2 in principal cells of the papilla is due to ectopic expression of the lacZ reporter gene, we used cell type-specific hybridization probes for cell discrimination in papillae of WT animals. As shown in Figure 4, principal cells were labeled with the Aqp2 probe, A-ICs with a probe showing adhesion receptor Gpr116 mRNA expression (Zaidman et al., 2020) and a pendrin probe for type B intercalated cells (B-ICs). Physiological expression of P2ry14 in the mouse kidney was restricted to the outer medulla (Figure 4A) and the renal papilla (Figure 4B). In the cortex, only single cells with a low but P2ry14-specific signal within collecting duct segments were detected when WT and KO tissues were compared (Supplementary Figure S2A). The probes for Gpr116 (type A-ICs) and pendrin (type B-ICs) showed localization of IC cells between Aqp2-positive principal cells of collecting ducts (Figure 4A; Supplementary Figure S2A). In the outer medulla a P2ry14-positive staining of Gpr116-positive IC cells was found indicating and confirming expression of P2ry14 in type A-ICs (Figure 4A) (Azroyan et al., 2015; Chen et al., 2017). In comparison, the P2ry14-staining of Gpr116-positive IC cells was lost in KO tissue (Supplementary Figure S2B). As described in the literature, type B-ICs are only present in the renal cortex but not in the medulla (for review see Al-Awqati et al., 2011). Consistently, pendrin expression was only detectable in the cortex (Supplementary Figure S2A). In the tip of the renal papilla, considerable P2ry14 staining was detectable in the collecting duct system in WT tissue. Most P2ry14-positive cells colocalized with Aqp2 showing expression of P2ry14 in collecting duct principal cells of the renal papilla. P2ry14-positive cells were also seen in the papilla tip-lining epithelia (Figure 4B). Interestingly, the presumably unspecific red background seen in the cortex and medulla of WT and KO kidney was absent in the papilla. Despite the lack of IC cells in the papilla tip, the Gpr116 probe showed an expression of Gpr116 (Figure 4B; Supplementary Figure S2B). In contrast to the expression in type A-ICs in the outer medulla, the papillary expression of Gpr116 was seen in the interstitial tissue surrounding the collecting ducts.

Our *in-situ* hybridization results confirmed the X-Gal and SPiDER-Gal experiments with KO animals showing that ß-galactosidase expression in KO kidneys perfectly reflects P2ry14 expression in WT kidneys and underscores that P2ry14 expression is not restricted to intercalated cells but is particularly high in principal cells of the renal papilla and in cells lining the papilla.

### Loss of P2ry14 alters the microscopic structure of the mouse kidney

The P2ry14 expression increases during maturation of the kidney after birth (Figure 2). To determine whether P2ry14 influences the development and structure of the kidney, we compared the histological structure of kidneys from WT and KO mice. Alcian blue- and PAS-stained sections displaying the entire kidney were prepared from kidneys of both sides. After manual delimitation of the different areas, the number of glomeruli and the size of different subareas (Supplementary Figure S2) were determined. The comparison of total and of distinct kidney areas revealed differences between WT and KO (Supplementary Table S3). The total kidney area was significantly larger in KO animals compared to WT animals (Supplementary Table S3). The ISOM and the IM/P showed no significant difference between WT and KO kidneys (Supplementary Table S3). However, significant differences were found for the cortex, the OSOM and the glomeruli number when referred to the cortex area. KO mice had a broader cortex relative to the total kidney area than WT mice. In contrast, the area of the OSOM was smaller in KO compared to WT mice. The number of glomeruli per cortex area was significantly lower in KO mice but the absolute number of glomeruli per kidney was not different (Supplementary Table S3). Our results indicate, that loss of P2ry14 in KO mice influences the kidney size and architecture resulting in broader cortex *versus* OSOM areas whereas the total number of glomeruli was not different.

### P2ry14-deficiency causes major transcriptomic changes in renal papillae

Previous results of Azroyan (Azroyan et al., 2015), our group (Meister et al., 2014) and of this study (see above) indicated that P2ry14 may play a role in kidney development and function. To expose the underlying mechanisms, we investigated transcriptomic changes in papillae of WT and KO female mice under regular chow and under a NaCl-enriched diet (salt challenging conditions). Thus, mice were split into four groups (WT/chow; KO/chow; WT/salt; KO/salt). As expected, genes that are characteristic for kidney papillae were expressed in all samples (Supplementary Table S4). Aqp2 and the subunit B1 of the proton transporting V-ATPase ATP6V1 (Lee et al., 2015; Clark et al., 2019; Zaidman et al., 2020) are among these genes. Furthermore, the data from KO mice showed a strong decrease in reads mapped to the P2ry14 gene confirming the successful knockout. Because only the coding exon of P2ry14 is deleted and the non-coding exons are still present in our mouse strain, reads of the 5′UTR still map to the P2ry14 gene allowing expression analysis even in KO samples (Supplementary Table S4 for FPKM values, Supplementary Tables S5, S6 for differential expression analysis).

The total number of differentially expressed genes between the different groups is shown in Supplementary Figure S3A. Interestingly, the number of differentially expressed genes were more dependent on the diet (chow vs. salt-enriched diet) than of the genotype as found with the principal component analysis (Supplementary Figure S3B). This is in line with a previous study showing the eminent influence of the diet on gene expression rather than of the genotype (Lede et al., 2017). Among those genes which are significantly regulated by salt in both WT and KO are GPCRs (Avpr2, Fzd1), B cell leukemia/lymphoma 6 (Bcl6), ATPases (Atp6v0a4, Atp6v0d2, Atp6v1g3), and arginase type II (Arg2), all known to show significant expression changes upon high-salt diet (Swanson et al., 2019). Gene ontology (GO) analysis of significantly expressed genes between WT and KO and the diets revealed only unspecific categories if significance of the false discovery rate is considered (Supplementary Table S7). However, if only significance of *p* values is considered, some categories related to specific kidney functions are found in both chow and salt diets as well as WT and KO including mitochondrial functions and lipid metabolism.

To identify genes significantly regulated by P2ry14, we extracted genes that were differentially expressed between WT and KO mice under chow as well as salt-enriched diet. This resulted in a total of 25 significantly changed transcripts. Of these, 19 genes were regulated concordantly. Examples include transcripts for proteins that are components of the extracellular matrix (e.g., decorin, fibulin-1, fibulin-7) and proteins involved in SM metabolism (e.g., serine palmitoyltransferase, small subunit b) (see Supplementary Table S8). Decorin is a proteoglycan involved in interactions with collagens and forms complexes with TGF-ß resulting in changes of TGF-ß signaling (Yamaguchi et al., 1990; Hildebrand et al., 1994). Fibulins are secreted glycoproteins with Ca^2+^-binding motifs interacting with different proteins and proteoglycans in the extracellular environment (Argraves et al., 1990). The serine palmitoyltransferase (SPT) is the rate-limiting enzyme in the *de-novo* biosynthesis of SM. It is a trimeric enzyme composed of two large and one small subunits. The large subunits are either SPTLC1 and SPTLC2 or SPTLC1 and SPTLC3. Full activity is conferred by an additional small subunit: SPTssa or SPTssb. Isoenzymes with a specific combination of subunits have preferences for different acyl-CoA species (Han et al., 2009). Strong downregulation of one of these small subunits points to possible changes in pathways involved in the biosynthesis of SM. Almost all subunits of the enzyme were expressed abundantly in the renal papilla except for the large subunit Sptlc3 (Supplementary Table S5). Our transcriptome approach revealed a significant downregulation of Sptssb in KO mice under both diets. The other subunits were not differentially expressed between WT and KO papillae (Supplementary Tables S5, S6).

**FIGURE 5:**
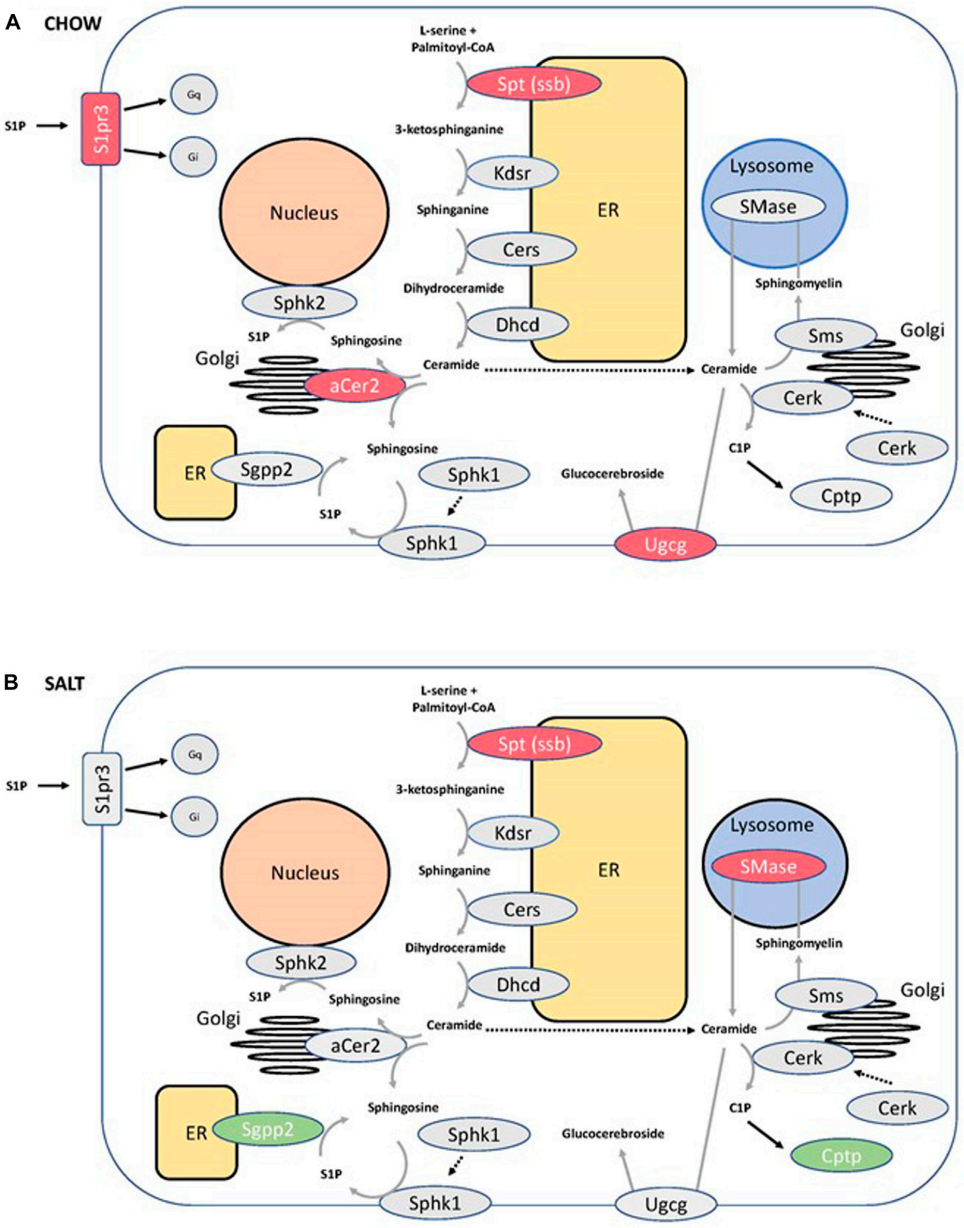
Changes in the sphingomyelin pathway in P2ry14-deficient mice. Several components involved in sphingomyelin pathway are shown. P2ry14 deficiency (KO) led to increased (green) and decreased (red) transcription of different genes compared to WT mice. Transcription changes between KO and WT under chow **(A)** and salt-enriched diet **(B)** are illustrated. For detailed statistical data of the RNA sequencing experiments see Supplementary Tables S5, S6. (aCer2: alkaline ceramidase 2; Cerk: ceramide kinase; Cers: ceramide synthase; Cptp: ceramide-1-phosphate-transfer protein; Dhcd: dihydroceramide desaturase; ER: endoplasmic reticulum; Kdsr: 3-ketodihydrosphingosine reductase; S1P: sphingosine-1-phosphate; S1pr3: sphingosine-1-phosphate receptor 3; Sgpp2: sphingosine-1-phosphate phosphatase 2; SMase: sphingomyelinase; Sms: sphingomyelin synthase; Sphk1,2: sphingosine kinase 1 and 2; Spt: serine palmitoyltransferase; ssb: small subunit b; Ugcg: UDP-glucose ceramide glucosyltransferase).

The differential expression of Sptssb prompted us to screen for additional changes in the expression of genes involved in SM metabolism and signaling. In chow-fed mice, downregulation of sphingosine-1-phosphate receptor 3, alkaline ceramidase 2 and UDP-glucose ceramide glucosyltransferase was observed in P2ry14 KO mice. Papilla samples of mice fed with a salt-enriched diet showed upregulation of sphingosine-1-phosphate phosphatase 2 (Sgpp2) and ceramide-1-phosphate transfer protein (Cptp) as well as downregulation of acid lysosomal sphingomyelin phosphodiesterase 1 (Smpd1) in P2ry14-deficient mice. Results are illustrated in Figure 5 (for statistical data see Supplementary Table S6).

To investigate the distribution of Spt subunits in different regions of the mouse kidney, we performed quantitative expression analysis using RT-qPCR on samples of three WT mice. The three large subunits of Spt were expressed in renal cortex, medulla and papilla but the expression levels differed substantially. Sptlc2 seems to be the dominant large subunit of Spt in the mouse kidney (ΔCq values to ß2-microglobulin: Sptlc1: cortex = 6.29 ± 0.25; medulla = 5.78 ± 0.43; papilla = 4.24 ± 0.88; Sptlc2: cortex = 1.86 ± 0.45; medulla = 2.24 ± 0.71; papilla = 1.20 ± 0.26; Sptlc3: cortex = 7.69 ± 0.31; medulla = 9.01 ± 1.47; papilla = 8.31 ± 0.24). The small regulatory subunits of Spt, Sptssa and Sptssb, were also expressed in all renal areas but the expression of Sptssb differed markedly between the different kidney regions. We found a considerable increase in expression of Sptssb from cortex to papilla. The expression of both regulatory subunits a and b in the kidney papilla was similar (ΔCq values to ß2 microglobulin: Sptssb: cortex = 9.53 ± 0.67; medulla = 6.96 ± 1.62; papilla = 0.64 ± 0.73; Sptssa: cortex = 2.70 ± 0.48; medulla = 2.57 ± 0.69; papilla = 0.74 ± 0.51).

### Renal sphingolipid metabolism in P2ry14-deficient mice

**FIGURE 6:**
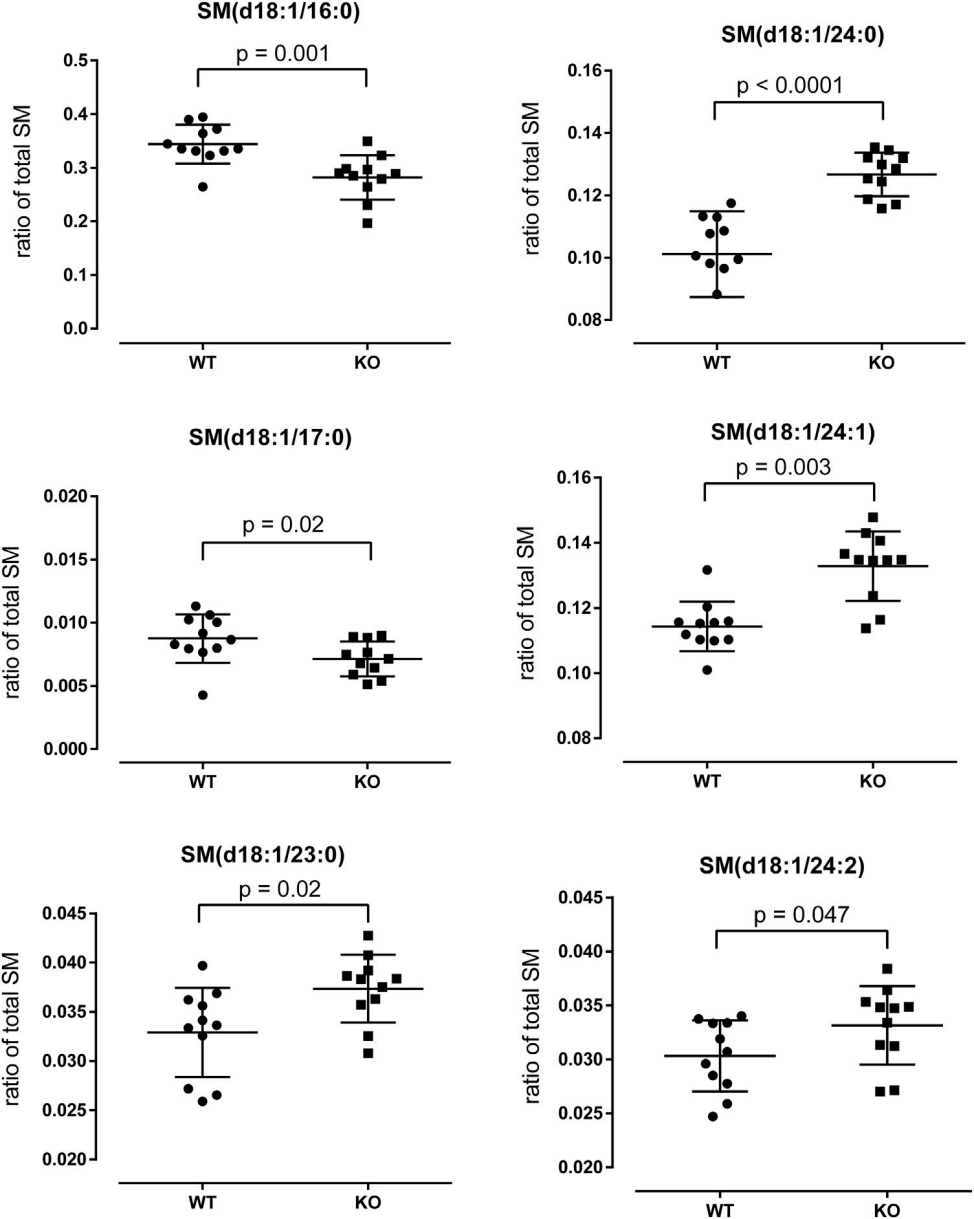
Analysis of sphingomyelin (SM) species in WT and KO papillae. WT and KO papillae were prepared, lipids were extracted by the MTBE method and HPTLC with subsequent ESI-IT MS measurements were performed (see Materials and Methods). Comparison of the relative amounts of different SM species (ratio of total SM; total SM = 1) of WT and KO papillae are shown. The numbers given in parentheses [e.g., SM(d18:1/16:0)] refer to the length of the two carbon chains and the number of double bounds. Statistical analysis was performed with GraphPad Prism 6 using the Mann-Whitney-U-test.

To study the biological significance of the downregulation of Sptssb in KO papillae, we collected papillae from WT and KO mice and performed SM analysis using HPTLC and ESI-IT MS measurements. The total amount of sphingomyelins (SM) was not significantly different between WT and KO mice (Supplementary Figure S4). Next, we analyzed the fatty acid chain composition of SM and found a number of significant differences (Figure 6). For example, we found a significant reduction of the SM(d18:1/16:0) species in KO mouse papillae whereas SM(d18:1/24:X) species were increased (Figure 6). P2ry14 deficiency resulted in a shift to SM with greater chain lengths compared to WT samples.

### Excretion of urine is decreased in knockout mice

To examine whether P2ry14 deletion results in changes in kidney function, we kept WT and KO mice in metabolic cages. Mice initially received regular chow diet (first phase) followed by challenging kidney function with a salt-enriched diet (second phase) after an adaptation period in between. Food and water consumption as well as excretion were recorded. After excluding invalid samples, a total of 16 mice per group were eligible for further analysis. The food intake was measured constantly over 72 h and did not differ significantly under both chow and salt diet between WT and KO mice (chow: WT = 4.63 ± 4.03 g; KO = 4.69 ± 4.27 g; *p* = 0.945 salt: WT = 3.6 ± 0.42 g; KO = 3.32 ± 0.92 g; *p* = 0.29). Similar results were seen with respect to water consumption. Although we found a tendency to lower water consumption in KO mice (chow: WT = 3.25 ± 1.14 ml; KO = 2.52 ± 1.33 ml; *p* = 0.28; salt: WT = 7.48 ± 1.93 ml; KO = 7.07 ± 6.06 ml; *p* = 0.09).

**FIGURE 7:**
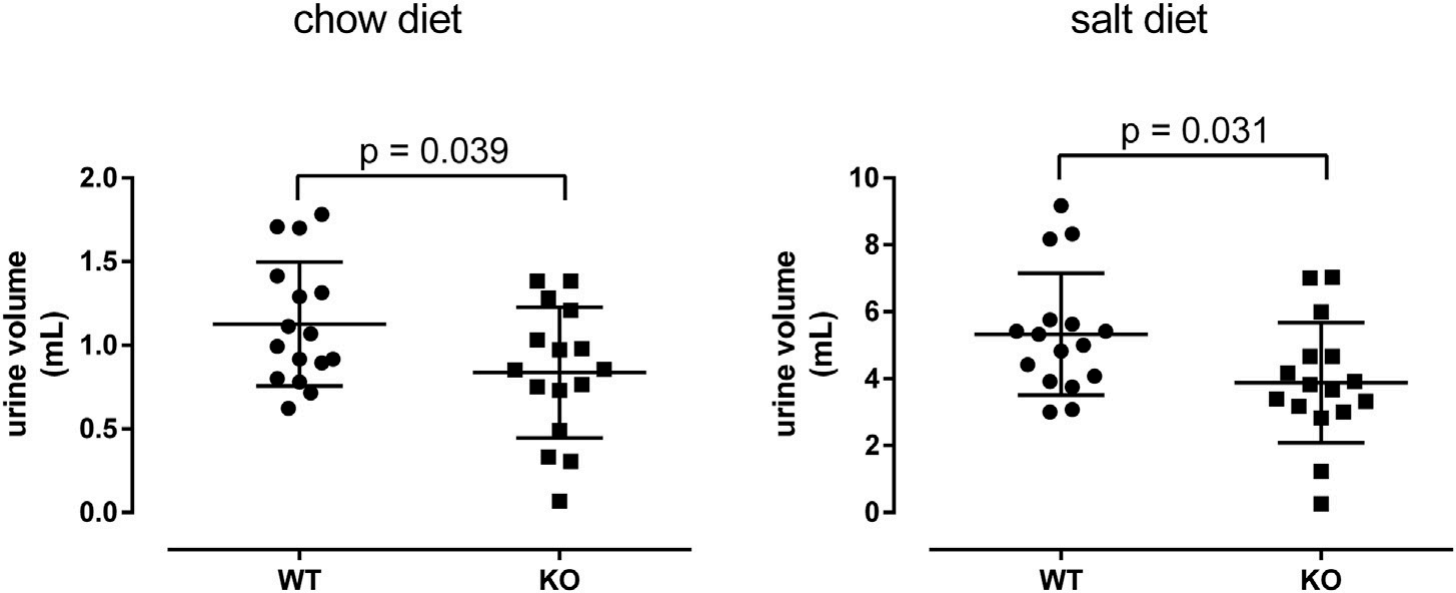
The daily urine volume is reduced in KO mice. WT and KO mice in metabolic cages received regular chow diet (first phase) followed by a salt-enriched diet (second phase) after an adaptation period in between. Daily urine excretion was recorded. The Mann-Whitney-U-test was used, n = 16 per group.

Feces and urine were collected and measured every 24 h. No difference in the amount of feces was found between WT and KO mice (chow: WT = 1.226 ± 0.397 g; KO = 1.363 ± 0.476 g; *p* = 0.220; salt: WT = 0.492 ± 0.086 g; KO = 0.453 ± 0.140 g; *p* = 0.355). However, we found the daily urine volume to be significantly lower in KO compared to WT mice. This was seen under chow as well as under salt-enriched diet (Figure 7). To see if this difference is due to a reduced kidney function, we measured and analyzed the decrease of FITC-inulin fluorescence in serum samples of four WT and seven KO mice (Qi et al., 2004; Qi und Breyer, 2009). We found no significant difference in fluorescence at any time point indicating no significant difference in kidney’s filtrating function between WT and KO mice (Supplementary Table S9). Furthermore, the reduced urine volume may result from different cAMP levels in the collecting ducts because cAMP is the main second messenger promoting Aqp2 plasma membrane insertion. However, the cAMP content in the papillae of WT and KO was not different between both genotypes (ratio cAMP content/protein concentration; chow: WT = 3.77 ± 2.80, n = 13; KO = 3.99 ± 1.97, n = 11; *p* = 0.76). This is in line with unchanged mRNA expressions of the vasopressin receptor type 2 (Avpr2), Gs protein (Gnas), adenylyl cyclases (Adcy4-7, 9), PKA (Prkaca, Prkacb), phosphodiesterases 3/4 (Pde3a, Pde3b, Pde4a), and Aqp2 signaling pathway between WT and KO (Supplementary Table S6). Furthermore, the urine osmolalities determined every 24 h over 3 days from WT and KO mice were not significantly different between the genotypes (chow: WT = 3612 ± 421 mosmol/kg, n = 13; KO = 3760 ± 820 mosmol/kg, n = 11; *p* = 0.88). This supports the above findings that a decreased reabsorption of water is not the cause of the reduced urine volumes in KO mice. Finally, we analyzed blood pH of WT and KO mice because P2ry14 is expressed in outer medullary type A-ICs, which are involved in acid-base regulation. No significant difference was detected between WT and KO mice, indicating that loss of P2ry14 expression in A-ICs has no prominent impact on blood pH levels (WT = 7.23 ± 0.07, n = 17; KO = 7.21 ± 0.10, n = 14; *p* = 0.85).

## Discussion

We used a knockout/lacZ-knockin mouse model to investigate the expression and function of P2ry14 in the kidney. We found that P2ry14 is highly expressed in the renal papilla (Figure 1) with increasing intensity towards the tip of the papilla. At birth, the expression of P2ry14 is rather low, but increases rapidly during postnatal development (Figure 2). Little is known about renal P2ry14 function. Azroyan and coworkers (2015) proposed a proinflammatory function of the receptor in the kidney. Consequently, genetical loss or pharmacological inhibition of P2ry14 turned out to be protective against acute kidney injury in a mouse model of ischemia reperfusion injury (Battistone et al., 2020). However, its proposed expression in ICs suggests additional function on basic kidney function parameters such as urine volume, GFR and blood pH, which have not been studied so far.

Using a mouse model with enhanced green fluorescent protein (EGFP) under the control of the promoter of the V-ATPase B1 gene, Azroyan and coworkers (2015) found a colocalization of P2ry14 in type A-ICs of the collecting ducts (Azroyan et al., 2015). ICs occur in the collecting ducts of the cortex and outer medulla (Roy et al., 2015). However, the nature and origin of the IC populations in the cortex and medulla seem to be different. Whereas in mice type B-ICs are only present in the cortex region, type A-ICs can be detected in the cortex and outer medulla and are not present in papilla. Interestingly, type A-IC populations in cortex and medulla have a different developmental origin and can change their phenotype to type B-ICs and back depending on the diet (reviewed in Al-Awqati et al., 2011; Iervolino et al., 2020). The obvious discrepancy of the distribution of principal cells, ICs and the strong P2ry14 expression in the kidney papilla prompted us to screen for further cell types expressing P2ry14. In KO animals, ß-galactosidase activity was used for X-Gal or SPiDER-Gal staining of cells. In contrast to X-Gal, SPiDER-Gal is a non-diffusible fluorescent dye and marks enzyme-expressing cells more accurate according to the manufacturer. In the renal papilla, we found ß-galactosidase expression in Aqp2-expressing principal cells and in cells lining the tip of the papilla (Figure 3C). *In-situ* hybridization in WT tissue using a P2ry14 probe and probes for pendrin (type B-ICs), Aqp2 (principal cells) and Gpr116 (type A-ICs) confirmed the expression of P2ry14 in type A-ICs of the outer medulla and principal cells of the papilla and cells lining the renal papilla (Figure 4). Studies in rats showed that the epithelial cells covering the tip of the renal papilla differ from the urothelium lining the kidney’s pelvis (Souza et al., 2018). To our best knowledge, no further characteristics of these epithelial cells are known. Several studies described the presence of adult kidney stem cells in the renal papilla of rats. Although not restricted to the terminal papilla, these stem cells seem to be enriched under the epithelial layer lining the renal papilla (Al-Awqati und Oliver, 2006; Oliver et al., 2009; Oliver et al., 2016). Whether P2ry14 is a characteristic feature of the mature epithelium lining the kidney papilla or if it is involved in the function of renal adult stem cells need to be investigated in future studies.

P2ry14 expression increases during postnatal kidney development (Figure 2). This may suggest a role of P2ry14 in the organogenesis of the kidney. Therefore, we analyzed the renal morphology. Our results showed significant larger kidneys and cortices and smaller OSOM areas in KO compared to WT mice (Supplementary Table S3). Although the absolute number of glomeruli was not different between WT and KO kidneys, the number of glomeruli in relation to the larger cortex or total kidney area was different. One can speculate that this reflects a compensatory hypertrophy of the cortex/kidney as shown in rats with unilateral nephrectomy (Pfaller et al., 1998). However, additional experiments are necessary to identify pathways regulated by P2ry14 that are involved in kidney development. Nevertheless, we clearly show that a constitutive loss of P2ry14 function has a developmental impact on renal morphology in mice (Supplementary Table S3). Currently, there is no human monogenetic disease related to inactivating mutations in P2RY14. Furthermore, P2RY14 does not belong to those GPCRs showing a low tolerance of inactivation (Schöneberg und Liebscher, 2021; Cao et al., 2022). Although there are studies on expression quantitative trait loci (eQTL) suggesting a P2RY14 association with asthma (Ferreira et al., 2017), to our best knowledge renal phenotypes have not been associated with DNA variants in the human P2RY14 gene.

Our phenotype analysis showed that loss of P2ry14 resulted in lower urine volumes under chow and salt diet (Figure 7) and a tendency to less water consumption under salt diet (see above). To differentiate between changes of glomerular filtration and downstream tubular mechanisms that regulate water homeostasis, we measured FITC-inulin clearance as a surrogate for glomerular filtration of WT and KO mice. Since the decline of FITC-inulin from blood was similar in both genotypes (Supplementary Table S9) we assume that mechanisms downstream of the glomerulus are crucial for reduced water excretion due to loss of functional P2ry14. However, urine osmolality and components of the vasopressin-mediated water reabsorption pathway were not significantly different between the genotypes. Although lower urine volumes are in line with a reduced water intake in the KO one would expect a higher urine osmolality in KO considering comparable excretion of osmolytes in WT and KO due to dietary intake. Currently, we do not have a proper explanation for this discrepancy but we consider the determined urine osmolality with caution because the already low volume of mouse urine collected over 24 h (1 ml) probably underwent significant evaporation. Furthermore, since it is almost impossible to prevent food remains and feces falling into the collected urine, these contaminations may have added osmolytes equalizing osmolalities between WT and KO. Future studies with repetitive measurements of spontaneous urine will study the concentration abilities of KO and WT kidneys in more detail.

Type A-ICs are proton secreting cells and play a decisive role in acid base homeostasis (Roy et al., 2015). The expression of P2ry14 in type A-ICs promoted us to measure blood pH of WT and KO mice but no significant differences were detected indicating only a minor influence of P2ry14 on basic kidney functions. Currently, we interpret the significant lower urine volumes in KO animals rather as a result of a lower water intake than a direct cellular impact of P2ry14 function on collecting duct-mediated water reabsorption. Otherwise, we cannot exclude that global deletion of P2ry14 influences kidney function by changes of extra-renal factors. For example, histology of different tissues using the ß-galactosidase expression in KO animals shows positive staining in the adrenal cortex which could have implications in hormone secretion and eventually kidney regulation (own, unpublished data). Moreover, high P2ry14 expression in the gastrointestinal tract and lung may also influence water homeostasis. Further studies are needed to evaluate specific functions of P2ry14 in other tissues to generate a holistic picture of P2ry14 function in mammals.

Considering the high expression of P2ry14 in the renal papilla, we collected individual papillae of WT and KO animals under regular conditions and kidney-challenging conditions (high salt-diet) and analyzed the transcriptome depending on the presence of P2ry14. Analysis revealed 409 differentially expressed genes between chow diet-fed WT and KO mice and 628 differentially expressed genes in the group of salt-enriched diet-fed animals. An almost 10-fold higher number of differentially expressed genes was detected comparing the diets within one genotype (Supplementary Figure S3A). Diet-dependent differences (salt-enriched semi-synthetic diet vs. chow diet) were more prominent than genotype-dependent differences, a finding which has been observed with other diets as well (Lede et al., 2017). Since the salt-enriched diet was a semi-synthetic diet, we found a great influence of the diet on gene expression. To identify genes and pathways strongly regulated by P2ry14 we filtered for genes differentially expressed under both diets and regulated concordantly. As a result only 19 genes including the GPCR Gpr171, Sptssb and the extracellular matrix proteins decorin, fibulin-1 and fibulin-7 were genotype-specifically regulated in renal papilla (Supplementary Table S8).

Gpr171 was upregulated in P2ry14-deficient mice. P2ry14 and Gpr171 as well as other structurally related receptors (Gpr87, P2ry12, P2ry13) cluster cluster in close proximity at chromosome 3 (Rossi et al., 2013). The cause of upregulation of Gpr171 remains unclear. One can speculate that it could be due to either compensation for the functional loss of P2ry14 (assuming redundant functions) or an unknown combined expression regulation of both genes. GPR171 seems to be involved in different neuronal and immune functions (Gomes et al., 2013; Bobeck et al., 2017; Fujiwara et al., 2021) but its relevance in renal function has not been studied yet. In-depth analysis of the functional interaction and expression regulation of these receptors or studies with single and double-KO mouse models may help in uncovering GPR171 function in kidney.

We also found downregulation of Sptssb in KO mice linking P2ry14 to SM metabolism. Serine palmitoyltransferase (SPT) is the rate-limiting enzyme in the synthesis of SM (Figure 5). It catalyzes the first step of the *de novo* synthesis of SM: the condensation reaction of an amino acid and fatty acyl-CoA, usually L-serine and palmitoyl-CoA resulting in 3-ketodihydrosphingosine. Downstream of SPT an abundance of enzymes modifies the generated long chain bases yielding a great variety of different SM species (Hannun and Obeid, 2011). In recent years, SM lost their image as molecules being only constituents of membranes. SM are involved in different cellular functions as signaling, lipid raft composition, influencing mitochondria, and enzymatic functions (Hannun and Obeid, 2008; Hannun and Obeid, 2018). Dysregulation of SM metabolism and changes in SM composition can be responsible for cardiovascular and metabolic diseases and cancer (Chatterjee, 1998; Holland und Summers, 2008; Kroll et al., 2020). Disturbances in SM metabolism in the kidney were found to play a critical role in chronic kidney diseases (Merscher und Fornoni, 2014; Abou et al., 2017; Zhang et al., 2019). Decreased expression of one of the regulating subunits of SPT (SPTssb; Supplementary Table S8) pointed to possible changes in SM metabolism. Because the active enzyme is a trimer of two large subunits (SPTLC1 and 2 or SPTLC1 and 3) and one regulating subunit (SPTssa or SPTssb) we investigated the regional distribution of the subunits in the kidney using qPCR on samples from renal cortex, medulla and papilla. SPTLC 1 and 2 are the dominant large subunits expressed in the mouse kidney. The regulating subunits were both abundantly expressed in the papilla region (see above). However, Sptssb shows a marked increase in expression from cortex towards the papilla. This suggests that Sptssb plays a distinct role in the renal papilla whereas Sptssa functions as a housekeeping subunit. As stated above, small subunits of SPT render the enzyme fully active. Furthermore, the small subunit included in the enzyme is crucial for substrate specificity (Han et al., 2009). SPT in microsomes prepared from yeast expressing different isoenzymes of SPT showed substrate specificity depending on the small subunit. SPT comprising small subunit a prefers C14 or C16 acyl-CoA whereas small subunit b containing SPT prefers C18. Microsomes of CHO LyB cells expressing SPTLC1/2 only showed substantial activity with C18-CoA as a substrate when transfected with SPTssa but not with SPTssb (Han et al., 2009). A certain SPTssb mutation, *Stellar (Stl)*, results in an increased affinity of SPT toward C18 fatty acyl-CoA resulting in accumulation of C20 long chain bases (LCBs) and neurodegeneration (Zhao et al., 2015). Therefore, we set out to investigate possible changes in SM species of P2ry14-deficient compared with WT papillae. We analyzed different SM species using HPTLC and ESI-IT MS. Knockout of P2ry14 resulted in a shift towards higher chain lengths (Figure 6). This is in line with previous results highlighting out the crucial role of small subunits of SPT with respect to substrate specificity (Han et al., 2009; Zhao et al., 2015). The overall content of SM in the papilla was unchanged between WT and KO mice (Supplementary Figure S4). In addition, we found more genes associated with SM metabolism and signaling differentially expressed between WT and KO with possible implications for signaling (S1pr3), sphingosine and sphingosine-1-phosphate (S1P) generation (aCer2) and glucocerebroside metabolism (Ugcg) (Figure 5A). Challenging the kidney with a salt-enriched diet changed the regulation of enzymes involved in SM metabolism (except for Sptssb) towards increased expression of two phosphatases involved in breakdown of the signaling molecules S1P and C1P (S1pp2, Cptp) and downregulation of the lysosomal sphingomyelinase (Figure 5B). The confirmation of changes in SM composition due to downregulation of Sptssb in P2ry14-deficient mice by mass spectrometry suggests a metabolic role of P2ry14 in kidney papillae. The specific pathways linking P2ry14 and SM metabolism may involve the G_i_ protein/adenylyl cyclase pathway. Thus, we propose P2ry14 to be a novel regulator of SM composition in the renal papilla.

In the light of a changed microscopic kidney structure, the differential expression of the extracellular matrix components fibulin-1, fibulin-7 and decorin was of particular interest. Upregulation of decorin and downregulation of fibulin-1 and -7 may be indicative for changes in extracellular matrix composition and interactions in the renal papilla. Little is known about the function of fibulin-1 and -7 in the kidney. Expression of fibulin-1 is decreased in renal cell carcinoma and suppresses progression of cancer (Xiao et al., 2013). Fibulin-7 is involved in processes of calcification (Tsunezumi et al., 2018). The renal function of decorin has been studied more thoroughly. Decorin seems to be increased in processes of kidney fibrosis and development of chronic kidney disease (Vleming et al., 1995; Stokes et al., 2000). However, its effect appears to be rather beneficial in terms of preventing apoptosis, inflammation and fibrosis (Schaefer et al., 2007). Presumably, the interaction of decorin and TGF-β is of great significance. Decorin inhibits TGF-β-signaling (Yamaguchi et al., 1990) thereby preventing accumulation of extracellular matrix and fibrosis (Border et al., 1992). The injection of decorin hampered the TGF-β-mediated accumulation of extracellular matrix components in a rat model of glomerulonephritis (Border et al., 1992). Similar results were observed by overexpressing decorin in rat skeletal muscle *in vivo* (Isaka et al., 1996). Furthermore, decorin seems to be involved in regenerative processes in the kidney (Sallustio et al., 2017). Decorin binds and neutralizes TGF-β which is thought to be a central pathway in tissue fibrosis (Isaka et al., 1996). Hypothetically, upregulation of decorin in P2ry14-deficient mice might mediate antifibrotic and nephroprotective effects by inhibiting the TGF-β-pathway. In the light of the protective effect of P2ry14 inhibition in a mouse model of acute kidney injury (Battistone et al., 2020) this might be a promising field of future research.

## Conclusion

We found P2ry14 to be strongly expressed in collecting duct principal cells of the renal papilla. Increasing P2ry14 expression during postnatal development and a changed renal morphology point to a role in organogenesis. Basic kidney functions such as urine volume production as well as SM metabolism are influenced by P2ry14. Eventually, our work encourage future research to further illuminate the function of P2ry14 in the kidney including its expression in a not yet exactly characterized epithelial cell type of the renal papilla and the possible involvement of P2ry14 in nephroprotection by regulation of decorin.

## Data Availability

The data presented in the study are deposited in the NCBI repository in BioProject (ID: PRJNA923283) under the following link: https://www.ncbi.nlm.nih.gov/bioproject/PRJNA923283.
