# Supplementary material for: Reduced urine volume and changed renal sphingolipid metabolism in P2ry14-deficient mice
Source: Front Cell Dev Biol. 2023 May 11;11:1128456. doi: 10.3389/fcell.2023.1128456 (PMC10213973; doi:10.3389/fcell.2023.1128456)
Supplement: Supplementary file 1 [file DataSheet1.zip › Supplementary Material/Supplementary Data Sheet.pdf]

Supplementary Information

Reduced urine volume and changed renal sphingolipid metabolism in P2ry14-deficient mice

Fabian Baalmann[1], Jana Brendler[2], Anne Butthof[1], Yulia Popkova[3], Kathrin M. Engel[3], Jürgen Schiller[3], Karsten Winter[2], Vera Lede[1], Albert Ricken[2], Torsten Schöneberg[1*], and Angela Schulz[1*]

[1] Rudolf Schönheimer Institute of Biochemistry, Faculty of Medicine, Leipzig University , 04103 Leipzig, Germany

[2] Institute of Anatomy, Faculty of Medicine, Leipzig University , 04103 Leipzig, Germany

[3] Institute of Medical Physics and Biophysics, Faculty of Medicine, Leipzig University , 04107 Leipzig, Germany

Correspondences should be addressed to:
*Angela Schulz and Torsten Schöneberg, Rudolf Schönheimer Institute of Biochemistry, Faculty of Medicine, Leipzig University , Johannisallee 30, 04103 Leipzig, Germany
e-mail: angela.schulz@medizin.uni-leipzig.de, schoberg@medizin.uni-leipzig.de

Keywords: P2ry14, kidney, papilla, intercalated cells, aquaporin-2, sphingolipids

A

B

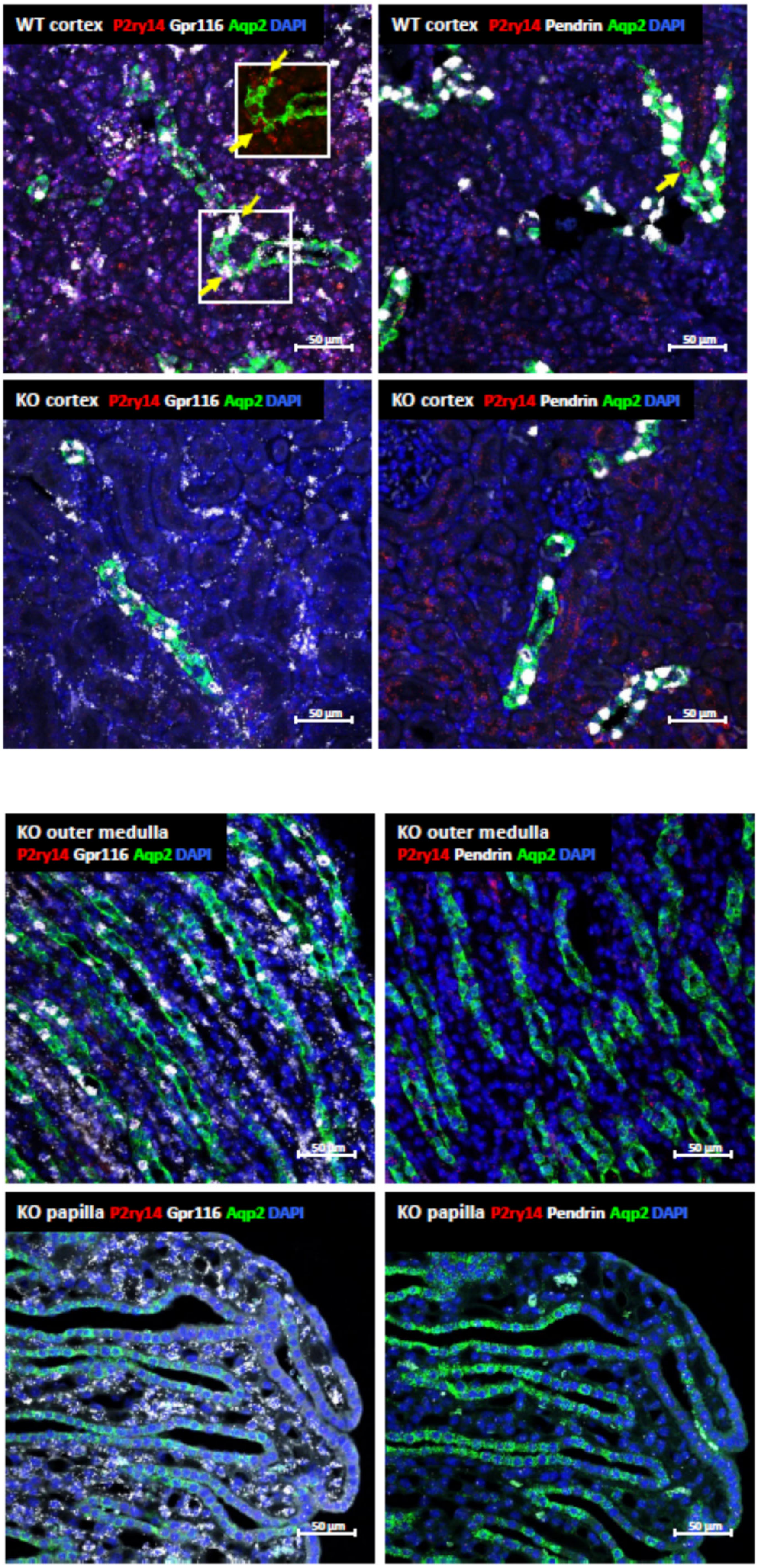
WT cortex P2ry14 Gpr116 Aqp2 DAPI
WT cortex P2ry14 Pendrin Aqp2 DAPI
KO cortex P2ry14 Gpr116 Aqp2 DAPI
KO cortex P2ry14 Pendrin Aqp2 DAPI
KO outer medulla
P2ry14 Gpr116 Aqp2 DAPI
KO outer medulla
P2ry14 Pendrin Aqp2 DAPI
KO papilla P2ry14 Gpr116 Aqp2 DAPI
KO papilla P2ry14 Pendrin Aqp2 DAPI
50 µm

C

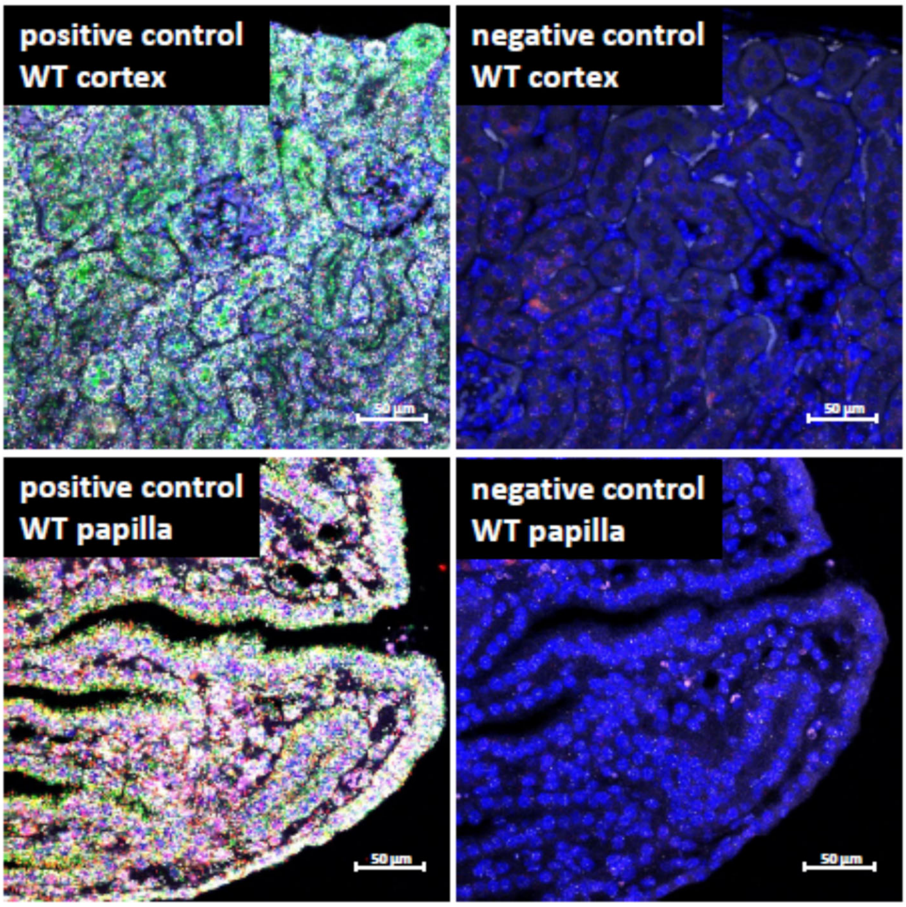

Suppl. Figure S1 Analysis of P2ry14 expressing cells in the kidney by *in-situ* hybridization.
For verification of P2ry14 expression in medulla and renal papilla of mice *in-situ* hybridization was performed in all kidney regions. To differentiate A-IC and B-IC cells from collecting duct principal cells, tissues were co-stained with probes for Gpr116 (A-ICs), pendrin (B-ICs) and Aqp2 (principal cells). Additionally, cell nuclei were stained with DAPI in blue. In WT cortex (**A**, upper panel), Aqp2 (green), Gpr116 (white, left panel) and pendrin (white, right panel) were expressed. P2ry14 expression was only detectable in single Gpr116-positive cells (yellow arrows) and hardly differentiable from the often unspecific red background staining similar to that observed in KO tissue (**A**, bottom figures, left and right). The inset in the upper left figure shows the same region and scale of the marked area in the figure without the signal of Gpr116 (white) for better visualization of the P2ry14 signal (red) in Gpr116-positive cells. (**B**) KO tissue from outer medulla was used as control (upper panel). In the papilla (bottom, left and right) the Gpr116 signal was restricted to interstitial tissue and pendrin expression was not observed. In addition, the punctuated, unspecific background staining found in other kidney regions of KO mice was not noted in the papilla region. To verify the specificity of the method, positive and negative probes were purchased from ACD. Results on kidney sections are shown in **C**. Positive probes stain the entire cortex and papilla tissue (left side) and negative probes failed to stain the tissue (right side). Details are described in *Material and Methods*.

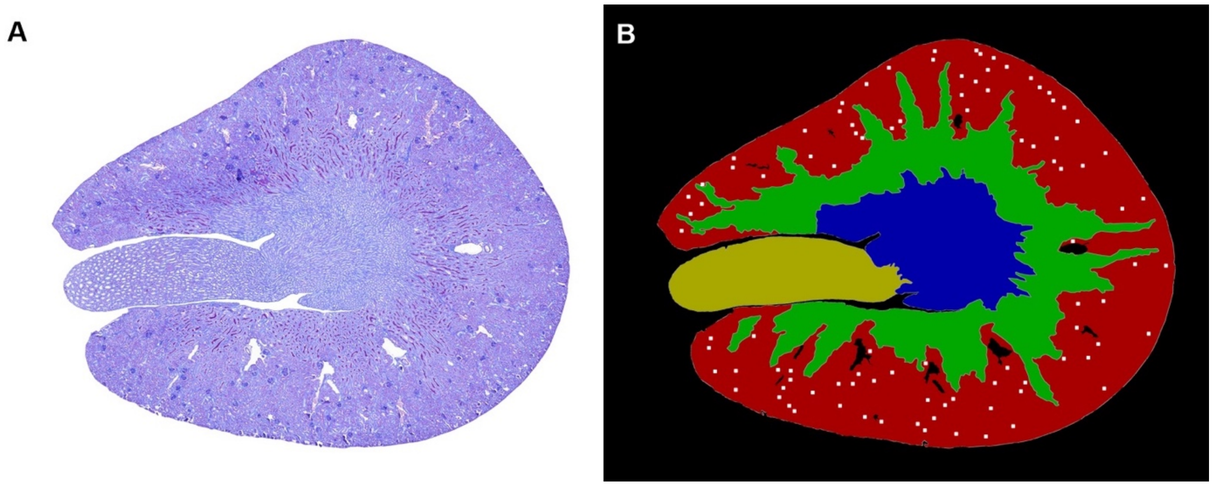

Suppl. Figure S2 Morphometric analysis of kidney sections.
To compare the microscopic architecture of WT and KO kidney sections, sections were stained with Alcian blue and PAS (**A**) (see *Materials and Methods*). For computational analysis, the different layers were manually encircled and color-coded (**B**). Cortex (red), outer stripe of the outer medulla -OSOM (green), inner stripe of the outer medulla -ISOM (blue) and inner medulla/papilla -IM/P (yellow). Analyses of area size and glomeruli number (white dots) were performed using the software Mathematica.

A

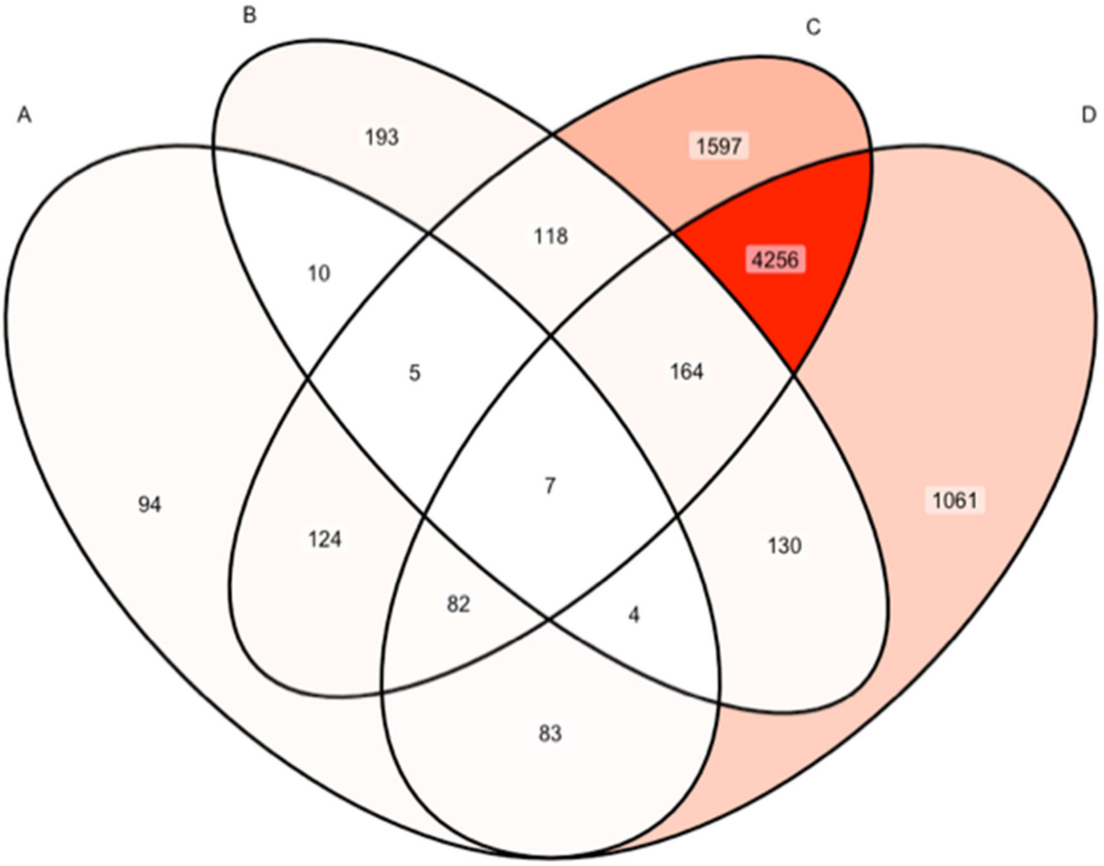

B

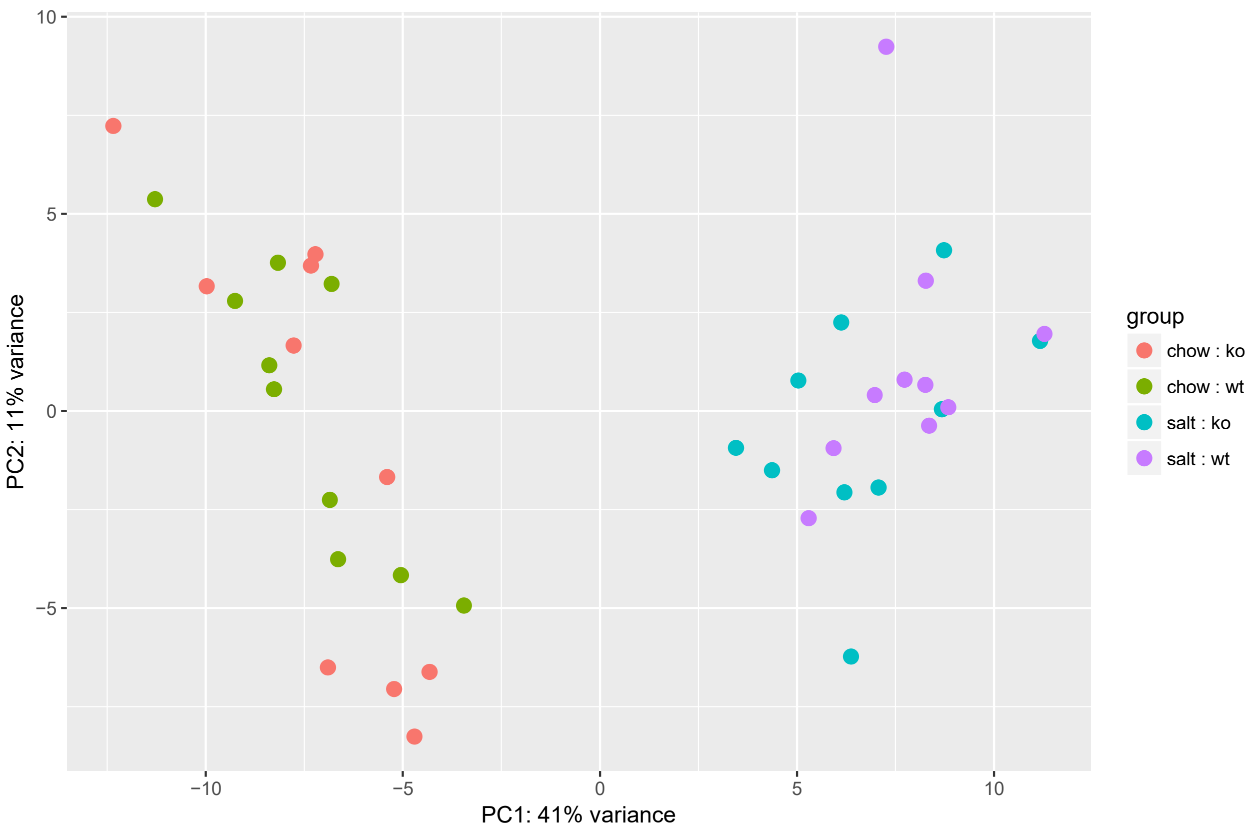

Suppl. Figure S3 Venn diagram and principal component analysis of differentially expressed genes between WT and KO depending on chow and salt diets.

A) The Venn diagram shows the overlap of differentially expressed genes between different conditions. A = WT vs. KO (chow diet), B = WT vs. KO (salt diet), C = chow vs. salt diet (WT), D = chow vs. salt diet (KO). The numbers depicted show the number of overlapping genes. B) Principal component (PC) analysis shows significant clustering depending on diet but not depending on genotypes.

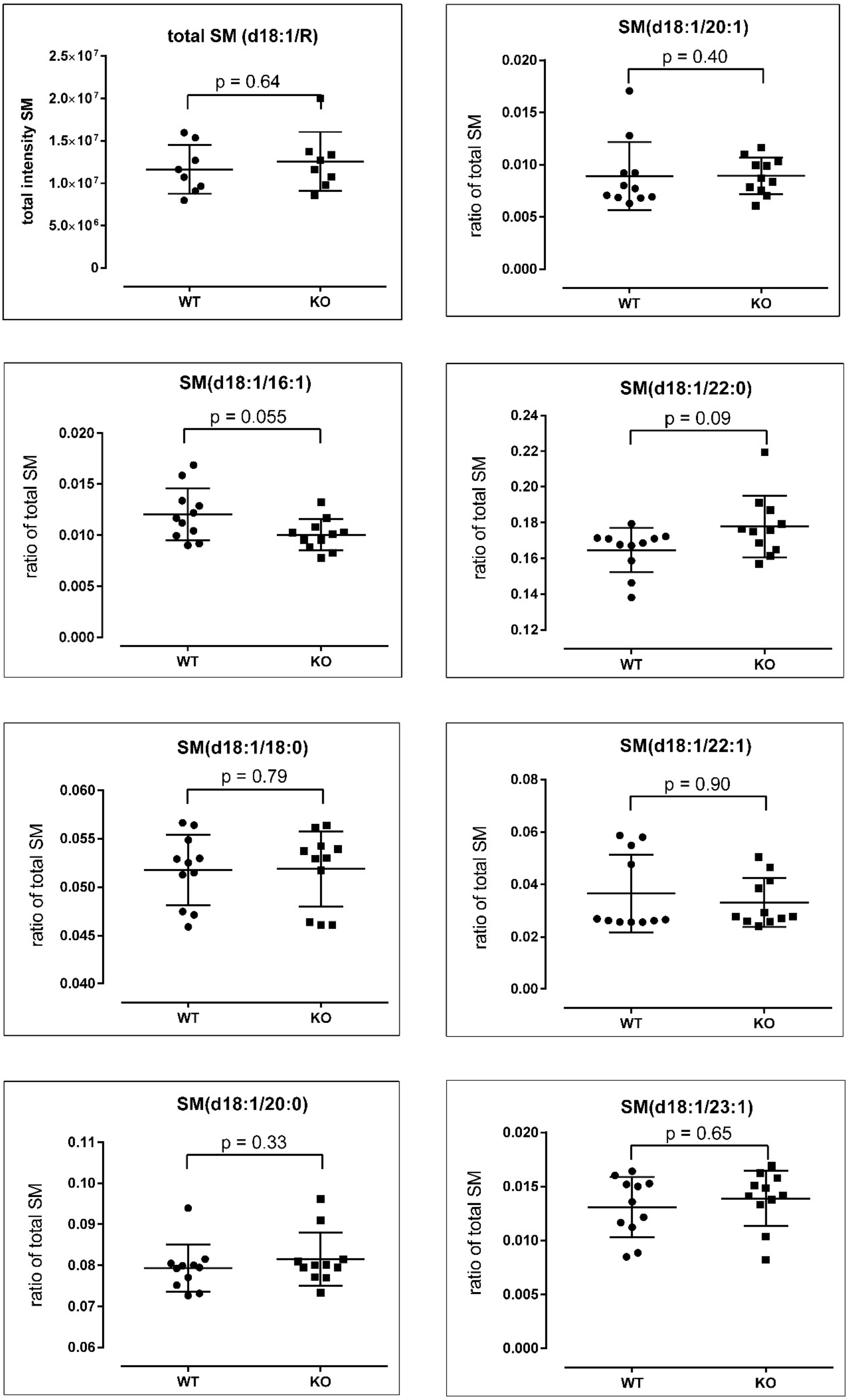

total SM (d18:1/R)
total intensity SM
p = 0.64
2.5×10⁷
2.0×10⁷
1.5×10⁷
1.0×10⁷
5.0×10⁶
0
WT
KO
SM(d18:1/20:1)
ratio of total SM
p = 0.40
0.020
0.015
0.010
0.005
0.000
WT
KO
SM(d18:1/16:1)
ratio of total SM
p = 0.055
0.020
0.015
0.010
0.005
0.000
WT
KO
SM(d18:1/22:0)
ratio of total SM
p = 0.09
0.24
0.22
0.20
0.18
0.16
0.14
0.12
WT
KO
SM(d18:1/18:0)
ratio of total SM
p = 0.79
0.060
0.055
0.050
0.045
0.040
WT
KO
SM(d18:1/22:1)
ratio of total SM
p = 0.90
0.08
0.06
0.04
0.02
0.00
WT
KO
SM(d18:1/20:0)
ratio of total SM
p = 0.33
0.11
0.10
0.09
0.08
0.07
0.06
WT
KO
SM(d18:1/23:1)
ratio of total SM
p = 0.65
0.020
0.015
0.010
0.005
0.000
WT
KO

Suppl. Figure S4 Total amount of sphingomyelins (SM) and detailed analysis of SM species in renal papillae.
WT and KO papillae were prepared, lipids were extracted by the MTBE method and HPTLC with subsequent ESI-IT MS measurements were performed (see *Materials and Methods*). Comparison of the relative amount of different SM species (ratio of total SM; total SM = 1) of WT and KO kidneys are shown. The numbers given in parentheses (e.g., SM(d18:1/16:0)) refer to the length of the two carbon chains and the number of double bounds. Analysis was done with GraphPad Prism 6 and for statistics, the Mann-Whitney-U-test was used.

Table S1 Composition of chow and salt diets
The diets were purchased from Sniff Spezialitäten GmbH, Soest, Germany.

Chow diet: total energy 16.2 MJ/kg

**Crude nutrients**	**%**	**Additives**	**per kG**
Crude protein	19.0	Vitamin A [IU/IE]	15,000
Crude fat	3.3	Vitamin D_3 [IU/IE]	1,100
Crude fiber	5.0	Vitamin E [mg]	110
Crude ash	6.4	Vitamin K [mg]	7
Starch	35.9		
Sugar	5.4	Copper [mg]	15
N-free extracts	54.6		

Salt diet: total energy 14.8 MJ/kg

**Crude nutrients**	**%**	**Additives**	**per kG**
Crude protein	21.1	Vitamin A [IU/IE]	15,000
Crude fat	5.1	Vitamin D_3 [IU/IE]	1,500
Crude fiber	5.0	Vitamin E [mg]	150
Crude ash	9.4	Vitamin K_3 [mg]	20
Starch	28.9	Vitamin C [mg]	30
Sugar	11.0	Copper [mg]	14
		Salt	4%

Supplemental Table S2. Primer sequences used in this study.

**gene**	**sense primer (5'->3')**	**antisense primer (5'->3')**
P2ry14	GCTGACTTTCTCATGGGCCT	GCCCAAAGAACACGATGCTG
SPTLC1 (flanking exon 3 / 4)	ACCACCCTGCTCTCAACTAC	CAGCAGCCCAAGGAAGTTAA
SPTLC2 (flanking exon 11 / 12)	CCCCGATCATTGAGTCCAGA	CCGGTGGCGAGAGTACTTTA
SPTLC3 (flanking exon 8 / 9)	TCTCGGGCTCAGGAGGATAT	GCTGCTACTACGGGACTCAT
SPTssa (flanking exon 1 / 2)	CTGGTCACTGCGCTCTACAT	GCATGAAGACGTAGCCAGTG
SPTssb (flanking exon 2 / 3)	AGCATCACCGAGTACCAAGC	GAGCATCGATTGTTCCCAAG

Supplemental Table S3. Morphometric analysis of kidney regions between WT and KO mice.
Different kidney regions were marked (cortex, outer stripe of the outer medulla -OSOM, inner stripe of the outer medulla -ISOM and inner medulla/papilla -IM/P) and areas analyzed and glomeruli counted automatically using Mathematica (Version 11.3, Wolfram Research Inc., Champaign, IL, USA). In total, n=18 kidney sections per group were summarized in the table. Significant differences (Student's t-Test) are marked with * p-value <0.05; ** p-value <0.01; *** p-value <0.001.

group	cortex (% of total)	OSOM (% of total)	ISOM (% of total)	IM/P (% of total)	number of glomeruli per section	total kidney section area (μm^2)	glomeruli/ cortex area	glomeruli/ total kidney area
WT	56.82 ± 2.85	21.82 ± 2.70	11.34 ± 1.06	10.02 ± 1.10	92.05 ± 11.20	13,329,454 ± 2,597,360	12.45 ± 1.97	7.08E-06 ± 1.2E-06
KO	58.76 ± 2.55 *	19.90 ± 2.83 *	10.95 ± 0.72	10.39 ± 1.18	94.77 ± 7.73	15,275,963 ± 1,887,628 *	10.69 ± 1.36 **	6.28E-06 ± 0.1E-06 *

Supplemental Table S4. FPKM values of all genes and groups.
The mean of the different groups and the individual values of the animals are given in the excel file Supplemental Table S4.xlsx.

Supplemental Table S5. Differential expression analysis of FPKM values of all genes and groups.
The data are given in the excel file Supplemental Table S5.xlsx

Supplemental Table S6. Filtered differential expression analysis of FPKM values of all genes and groups.
Filtering was conducted by including all genes of Suppl. Table S5 but excluding genes with FPKM cut off values <1 in at least half of the samples. Data are given in the excel file Supplemental Table S6.xlsx

Supplemental Table S7. GO categories of differentially expressed genes between WT and KO papillae respective of the diet.

Chow

GO-Category	N Genes	Direction	P-Value	FDR
STRUCTURAL_CONSTITUENT_OF_RIBOSOME	80	Up	4.17E+06	0,00006
MITOCHONDRIAL_INNER_MEMBRANE	75	Up	0,00004	0,02739
ORGANELLE_INNER_MEMBRANE	84	Up	0,00006	0,02739
RNA_POLYMERASE_ACTIVITY	16	Up	0,00046	0,14506
MITOCHONDRIAL_MEMBRANE_PART	60	Up	0,00074	0,14506
NADH_DEHYDROGENASE_COMPLEX	16	Up	0,00080	0,14506
MITOCHONDRIAL_RESPIRATORY_CHAIN_COMPLEX_I	16	Up	0,00080	0,14506
RESPIRATORY_CHAIN_COMPLEX_I	16	Up	0,00080	0,14506
PROTEASOME_COMPLEX	24	Up	0,00145	0,23381
SEQUENCE_SPECIFIC_DNA_BINDING	45	Down	0,00194	0,26621
MITOCHONDRIAL_RESPIRATORY_CHAIN	26	Up	0,00201	0,26621
SMALL_NUCLEAR_RIBONUCLEOPROTEIN_COMPLEX	25	Up	0,00220	0,26688
MITOCHONDRIAL_MEMBRANE	98	Up	0,00352	0,39355
PHOSPHATE_TRANSMEMBRANE_TRANSPORTER_ACTIVITY	9	Down	0,00382	0,39652
MITOCHONDRIAL_ENVELOPE	108	Up	0,00527	0,45645
EXTRACELLULAR_SPACE	138	Down	0,00530	0,45645
**BODY_FLUID_SECRETION**	12	Down	0,00534	0,45645
INOSITOL_OR_PHOSPHATIDYLINOSITOL_KINASE_ACTIVITY	20	Down	0,00651	0,52618
PRODUCTION_OF_MOLECULAR_MEDIATOR_OF_IMMUNE_RESP ONSE	7	Down	0,00716	0,54311
**LIPID_KINASE_ACTIVITY**	14	Down	0,00747	0,54311
VOLTAGE_GATED_POTASSIUM_CHANNEL_ACTIVITY	10	Down	0,00953	0,62890
U12_DEPENDENT_SPLICEOSOME	16	Up	0,00977	0,62890

EXTRACELLULAR_MATRIX_STRUCTURAL_CONSTITUENT	34	Down	0,00995	0,62890
**REGULATION_OF_BODY_FLUID_LEVELS**	46	Down	0,01219	0,73821
MITOCHONDRIAL_PART	179	Up	0,01377	0,80090
**ICOSANOID_METABOLIC_PROCESS**	29	Down	0,01445	0,80404
HUMORAL_IMMUNE_RESPONSE	15	Up	0,01565	0,80404
**THIOLESTER_HYDROLASE_ACTIVITY**	17	Down	0,01634	0,80404
CATION_CHANNEL_ACTIVITY	45	Down	0,01762	0,80404
POTASSIUM_CHANNEL_ACTIVITY	13	Down	0,01778	0,80404
CELL_FATE_COMMITMENT	13	Down	0,01794	0,80404
MONOOXYGENASE_ACTIVITY	22	Down	0,01870	0,80404
AMINE_RECEPTOR_ACTIVITY	5	Down	0,01887	0,80404
STRUCTURE_SPECIFIC_DNA_BINDING	66	Down	0,01989	0,80404
INORGANIC_ANION_TRANSPORT	15	Down	0,02105	0,80404
ION_CHANNEL_ACTIVITY	67	Down	0,02171	0,80404
COLLAGEN	32	Down	0,02208	0,80404
**MONOCARBOXYLIC_ACID_METABOLIC_PROCESS**	109	Down	0,02384	0,80404
STRUCTURAL_MOLECULE_ACTIVITY	225	Up	0,02388	0,80404
EXTRACELLULAR_REGION	285	Down	0,02443	0,80404
PROTEASE_INHIBITOR_ACTIVITY	23	Down	0,02475	0,80404
GROWTH_FACTOR_BINDING	27	Down	0,02494	0,80404
RESPONSE_TO_HEAT	13	Up	0,02506	0,80404
**FATTY_ACID_METABOLIC_PROCESS**	82	Down	0,02594	0,80404
DNA_DIRECTED_RNA_POLYMERASE_COMPLEX	19	Up	0,02599	0,80404
RNA_POLYMERASE_COMPLEX	19	Up	0,02599	0,80404
NUCLEAR_DNA_DIRECTED_RNA_POLYMERASE_COMPLEX	19	Up	0,02599	0,80404
GATED_CHANNEL_ACTIVITY	51	Down	0,02681	0,81226
EXTRACELLULAR_REGION_PART	221	Down	0,02752	0,81674
OUTER_MEMBRANE	27	Down	0,02934	0,85334
POSITIVE_REGULATION_OF_DNA_METABOLIC_PROCESS	7	Down	0,03093	0,88181
GROWTH	84	Down	0,03199	0,89311
CARBONATE_DEHYDRATASE_ACTIVITY	7	Down	0,03330	0,89311
PROTON_TRANSPORTING_TWO_SECTOR_ATPASE_COMPLEX	16	Up	0,03371	0,89311
RESPONSE_TO_TEMPERATURE_STIMULUS	22	Up	0,03378	0,89311
NEGATIVE_REGULATION_OF_ANGIOGENESIS	20	Down	0,03528	0,91598
SPLICEOSOME	59	Up	0,03679	0,92873
ACTIVATION_OF_PROTEIN_KINASE_ACTIVITY	30	Up	0,03744	0,92873
POSITIVE_REGULATION_OF_NUCLEOBASENUCLEOSIDENUCLEOTIDE_AND_NUCLEIC_ACID_METABOLIC_PROCESS	162	Down	0,03791	0,92873
SINGLE_STRANDED_DNA_BINDING	40	Down	0,03832	0,92873
REGULATION_OF_HEART_CONTRACTION	28	Down	0,03965	0,92900
SUBSTRATE_SPECIFIC_CHANNEL_ACTIVITY	71	Down	0,04013	0,92900
TRANSLATION	193	Up	0,04102	0,92900
**GLYCOSPHINGOLIPID_METABOLIC_PROCESS**	12	Down	0,04162	0,92900
REGULATION_OF_ENDOTHELIAL_CELL_PROLIFERATION	8	Up	0,04192	0,92900
G1_S_TRANSITION_OF_MITOTIC_CELL_CYCLE	26	Down	0,04217	0,92900
METALLOEXOPEPTIDASE_ACTIVITY	12	Up	0,04441	0,94688
VOLTAGE_GATED_CHANNEL_ACTIVITY	31	Down	0,04468	0,94688
G2_M_TRANSITION_OF_MITOTIC_CELL_CYCLE	10	Down	0,04504	0,94688
PROTEIN_AMINO_ACID_LIPIDATION	26	Up	0,04659	0,94688
**LIPOPROTEIN_BIOSYNTHETIC_PROCESS**	26	Up	0,04659	0,94688
NUCLEAR_SPECK	12	Up	0,04689	0,94688
RIBOSOME	39	Up	0,04835	0,95995
NEGATIVE_REGULATION_OF_INTRACELLULAR_TRANSPORT	8	Up	0,04887	0,95995
DEVELOPMENTAL_GROWTH	21	Down	0,04952	0,95995

salt

**GO-Category**	**N Genes**	**Direction**	**P Value**	**FDR**
ENDOPLASMIC_RETICULUM_MEMBRANE	95	Down	0,00058	0,43098
ENDOPLASMIC_RETICULUM_PART	109	Down	0,00064	0,43098
NUCLEAR_ENVELOPE_ENDOPLASMIC_RETICULUM_NETWORK	105	Down	0,00152	0,43098

DNA_HELICASE_ACTIVITY	22	Up	0,00168	0,43098
ATP_DEPENDENT_HELICASE_ACTIVITY	30	Up	0,00225	0,43098
HELICASE_ACTIVITY	51	Up	0,00230	0,43098
ENDOPLASMIC_RETICULUM	326	Down	0,00232	0,43098
EXOCYTOSIS	24	Down	0,00237	0,43098
CHEMOKINE_ACTIVITY	15	Down	0,00441	0,60402
CHEMOKINE_RECEPTOR_BINDING	15	Down	0,00441	0,60402
ATP_DEPENDENT_RNA_HELICASE_ACTIVITY	20	Up	0,00525	0,60402
G_PROTEIN_COUPLED_RECEPTOR_BINDING	20	Down	0,00552	0,60402
CALCIUM_ION_BINDING	100	Down	0,00554	0,60402
ENDOPLASMIC_RETICULUM_LUMEN	18	Down	0,00615	0,60402
OLIGOSACCHARYL_TRANSFERASE_COMPLEX	10	Down	0,00623	0,60402
CONTRACTILE_FIBER	19	Down	0,00866	0,74044
CONTRACTILE_FIBER_PART	19	Down	0,00866	0,74044
RNA_DEPENDENT_ATPASE_ACTIVITY	22	Up	0,01305	0,99762
**FATTY_ACID_BETA_OXIDATION**	21	Down	0,01361	0,99762
**SECRETORY_PATHWAY**	83	Down	0,01668	0,99762
ACTIN_POLYMERIZATION_AND_OR_DEPOLYMERIZATION	27	Down	0,01848	0,99762
GENERAL_RNA_POLYMERASE_II_TRANSCRIPTION_FACTOR_ACTIVITY	35	Up	0,01855	0,99762
**LIPASE_ACTIVITY**	28	Down	0,01940	0,99762
REGULATION_OF_HEART_CONTRACTION	28	Down	0,02053	0,99762
NEGATIVE_REGULATION_OF_MYELOID_CELL_DIFFERENTIATION	15	Up	0,02297	0,99762
TRANSCRIPTION_INITIATION_FROM_RNA_POLYMERASE_II_PROMOTER	31	Up	0,02323	0,99762
SPERMATID_DIFFERENTIATION	19	Down	0,02357	0,99762
PROTEASE_INHIBITOR_ACTIVITY	23	Down	0,02368	0,99762
RNA_HELICASE_ACTIVITY	25	Up	0,02481	0,99762
INTRAMOLECULAR_OXIDOREDUCTASE_ACTIVITY	19	Down	0,02694	0,99762
CYTOKINE_AND_CHEMOKINE_MEDIATED_SIGNALING_PATHWAY	26	Up	0,02838	0,99762
ACID_AMINO_ACID_LIGASE_ACTIVITY	67	Up	0,02930	0,99762
SERINE_TYPE_ENDOPEPTIDASE_INHIBITOR_ACTIVITY	15	Down	0,02953	0,99762
SPINDLE_POLE	16	Down	0,02976	0,99762
**FATTY_ACID_OXIDATION**	27	Down	0,03088	0,99762
UBIQUITIN_PROTEIN_LIGASE_ACTIVITY	59	Up	0,03090	0,99762
**SECRETION_BY_CELL**	106	Down	0,03162	0,99762
SPERMATID_DEVELOPMENT	17	Down	0,03219	0,99762
EPITHELIAL_CELL_DIFFERENTIATION	7	Up	0,03246	0,99762
ACETYLGALACTOSAMINYLTRANSFERASE_ACTIVITY	17	Down	0,03302	0,99762
STRUCTURAL_CONSTITUENT_OF_MUSCLE	28	Down	0,03428	0,99762
NADH_DEHYDROGENASE_COMPLEX	16	Down	0,03445	0,99762
MITOCHONDRIAL_RESPIRATORY_CHAIN_COMPLEX_I	16	Down	0,03445	0,99762
RESPIRATORY_CHAIN_COMPLEX_I	16	Down	0,03445	0,99762
LOCOMOTORY_BEHAVIOR	47	Down	0,03627	0,99762
**PHOSPHOLIPASE_ACTIVITY**	24	Down	0,03794	0,99762
INORGANIC_ANION_TRANSPORT	15	Up	0,03799	0,99762
ORGANELLAR_SMALL_RIBOSOMAL_SUBUNIT	11	Down	0,03949	0,99762
MITOCHONDRIAL_SMALL_RIBOSOMAL_SUBUNIT	11	Down	0,03949	0,99762

SMALL_RIBOSOMAL_SUBUNIT	11	Down	0,03949	0,99762
MEIOTIC_RECOMBINATION	10	Up	0,04061	0,99762
REGULATION_OF_JNK_CASCADE	22	Up	0,04070	0,99762
NUCLEOTIDYLTRANSFERASE_ACTIVITY	47	Down	0,04105	0,99762
G_PROTEIN_SIGNALING_ADENYLATE_CYCLASE_ACTIVATING_PATHWAY	8	Up	0,04106	0,99762
ADENYLATE_CYCLASE_ACTIVATION	8	Up	0,04106	0,99762
SMALL_CONJUGATING_PROTEIN_LIGASE_ACTIVITY	61	Up	0,04310	0,99762
LIGASE_ACTIVITY_FORMING_CARBON_NITROGEN_BONDS	77	Up	0,04323	0,99762
**SECRETION**	171	Down	0,04375	0,99762
OXIDOREDUCTASE_ACTIVITY_GO_0016706	11	Down	0,04478	0,99762
MITOCHONDRIAL_INNER_MEMBRANE	75	Down	0,04488	0,99762
SMALL_PROTEIN_CONJUGATING_ENZYME_ACTIVITY	62	Up	0,04635	0,99762
ER_GOLGI_INTERMEDIATE_COMPARTMENT	30	Down	0,04952	0,99762

Supplemental Table S8. Differentially expressed genes between WT and KO papillae irrespective of the diet.

Genes that are upregulated in KO mice are shown in green, downregulated genes are shown in grey.

Gene name	Description	Chow (log_2foldchange)	Chow (p-value)	Salt (log_2foldchange)	Salt (p-value)
P2ry14	purinergic receptor P2Y, G-protein coupled 14	-2.85291624	3.77E-21	-3.145685705	1.04E-82
Gpr171	G protein-coupled receptor 171	2.803513641	1.11E-08	4.676484604	1.72E-19
SPTssb	serine palmitoyltransferase. small subunit B	-0.731624055	9.02E-08	-0.661690887	2.40E-06
Tmem144	transmembrane protein 144	1.102658709	1.85E-06	0.8545628	9.82E-07
Ccdc107	coiled-coil domain containing 107	0.290194675	0.001382703	-0.231612004	0.044196224
Mme	membrane metallo endopeptidase	0.581509158	0.002960825	0.6508619	0.000447952
Medag	mesenteric estrogen dependent adipogenesis	-0.89820659	0.004862181	-0.515438804	0.03681081
Dcn	decorin	0.42691941	0.004965802	0.34884958	0.029819113
Mbnl1	muscleblind like splicing factor 1	-0.238055081	0.00510821	-0.352545758	0.004434732
Fam13c	family with sequence similarity 13, member C	-0.38859478	0.005728455	-0.276346909	0.013537621
Gm9967	predicted gene 9967	0.495024645	0.013011394	0.315341156	0.027261129
Gimap5	GTPase. IMAP family member 5	0.368775385	0.013642831	0.248011475	0.019718652
Hectd2	HECT domain E3 ubiquitin protein ligase 2	-0.514150856	0.01493174	0.480880562	0.019075977
Snf8	SNF8, ESCRT-II complex subunit, homolog (S, cerevisiae)	0.16806548	0.019874458	-0.130104587	0.032212395
Rian	RNA imprinted and accumulated in nucleus	0.372970581	0.021060786	0.502897947	0.037413272
BC031181	cDNA sequence BC031181	0.154533999	0.02106481	-0.112049504	0.038464052
Xlr3b	X-linked lymphocyte-regulated 3B	-0.829080152	0.021152634	-0.639190164	0.002267576
Fbln7	fibulin 7	-0.246396503	0.027664498	-0.252970758	0.022348644
Psmb1	proteasome (prosome. macropain) subunit, beta type 1	0.255935708	0.030327697	-0.248825064	0.023876772
Serpina1a	serine (or cysteine) peptidase inhibitor, clade A, member 1A	-0.69204677	0.034583459	-0.772762244	0.024811915
Fcgbp	Fc fragment of IgG binding protein	0.256127126	0.036233561	-0.387305927	0.033315874

Gm7008	predicted gene 7008	0.376409628	0.037158553	0.50185095	0.009566197
Rbm15	RNA binding motif protein 15	0.3757248	0.037990423	0.334691453	0.037719292
Fbln1	fibulin 1	-0.39083227	0.041119303	-0.411715679	0.00318587
Ick	intestinal cell kinase	-0.16318309	0.044182161	-0.17011376	0.046643345
AA986860	expressed sequence AA986860	0.257795451	0.048688637	0.280948736	0.008698086

Supplemental Table S9. Relative inulin clearance measurement with FITC-labelled inulin.
Peak fluorescence at 2 min after injection was set 100% (=1) and relative fluorescence at other time points was calculated.

**time** (min)	**WT** (mean)	**KO** (mean)	**difference**	**p-value**
2 (baseline)	1	1	0	1
4	0.6613	0.6303	0.03096	0.72468297
6	0.5598	0.5796	-0.01982	0.69875128
8	0.5583	0.4591	0.09911	0.25929217
10	0.3765	0.4571	-0.08064	0.30466419
15	0.3438	0.3083	0.03546	0.22514192
30	0.243	0.238	0.005	0.84799326
